# Family physicians’ intention to support women in making informed decisions about breast cancer screening with mammography: a cross-sectional survey

**DOI:** 10.1186/s13104-015-1608-8

**Published:** 2015-11-10

**Authors:** Lawrence-Ndoh Kiyang, Michel Labrecque, Florence Doualla-Bell, Stéphane Turcotte, Céline Farley, Myrtha Cionti Bas, Johanne Blais, France Légaré

**Affiliations:** Hôpital Saint-Francois d’Assise, Centre Hospitalier Universitaire de Québec Research Centre, 10 Rue de l’Espinay, Québec, QC G1L 3L2 Canada; Department of Family and Emergency Medicine, Université Laval, 1050, avenue de la Médecine, Pavillon Ferdinand-Vandry, Bureau 4617, Québec, QC G1V 0A6 Canada; Surgery Strategic Clinical Network, Alberta Health Services, Room 101, Materiel Management Centre, Royal Alexandra Hospital, 10240 Kingsway Avenue NW, Edmonton, AB T5H 3V9 Canada; Institut National de Santé Publique du Québec, 190, boulevard Crémazie Est, Montréal, QC H2P 1E2 Canada; Department of Social and Preventive Medicine, École de santé publique, Université de Montréal, C.P. 6128, Succursale Centre-ville, Montréal, QC H3C 3J7 Canada

**Keywords:** Breast cancer, Screening, Informed decision-making, Theory of Planned Behavior, Survey

## Abstract

**Background:**

The net benefits of routine breast cancer screening with mammography have been questioned, and there is evidence to indicate that supporting women to make an informed decision about breast cancer screening with mammography is preferable. The aims of this study were to assess the intention of family physicians to provide women with this support and the determinants of this intention, and to identify factors that might influence family physicians adopting this behavior.

**Methods:**

Family physicians from the province of Quebec, Canada, attending a 45-min lecture on informed decision making and cancer screening were asked to complete a questionnaire after the lecture regarding their intention to adopt the behavior. The questions, based on the Theory of Planned Behavior, measured physicians’ intention and its determinants (attitude, perceived behavioral control, and socio-professional norm) regarding supporting women to make informed decisions about breast cancer screening with mammography. Open-ended questions were also used to explore complementary factors influencing their intention.

**Results:**

Out of 800 questionnaires distributed, 301 (38 %) were returned and 288 were included in data analysis. The mean ± standard deviation and median score for intention were respectively 1.9 ± 1.2 and 2.0 on a 6-point Likert scale (−3 to +3). Perceived behavioral control was the variable most strongly associated with intention (high versus low score, odds ratio = 15.7, 95 % CI 6.7–36.6), followed by attitude (high versus low score, odds ratio = 7.5, 95 % CI 3.3–16.8), then social norm (high versus low score, odds ratio = 5.8, 95 % CI 2.6–12.9). The most-reported barrier to adopting the behavior was time constraints (41 %) while the most-reported facilitator was availability of relevant decision support tools (29 %).

**Conclusions:**

Respondents showed strong intention to support women in informed decision-making about breast cancer screening, the strongest predictor being perceived behavioral control. These results could contribute to training physicians to integrate this behavior into their practices and to designing relevant decision support tools.

**Electronic supplementary material:**

The online version of this article (doi:10.1186/s13104-015-1608-8) contains supplementary material, which is available to authorized users.

## Background

Many developed countries have national breast cancer screening programs based on mammography. In 1998, the Quebec Ministry of Health and Social Services (MHSS), Canada, began recommending that all women aged 50–69 participate in the Quebec Breast Cancer Screening Program (QBCSP). Their slogan was “Breast cancer screening saves lives”. However, when controversies arose about the net benefit of breast cancer screening with mammography [1–3] and new scientific evidence emerged about the benefits of informed/shared decision making regarding cancer screening [4–7], the MHSS revised its policy and the QBCSP slogan was replaced by “Screening for breast cancer: a choice that belongs to you” [8]. This approach emphasizes informed decision making, defined as occurring when an individual understands the disease or condition being addressed and understands what the clinical service involves, including benefits, risks, limitations, alternatives, and uncertainties; has considered his or her preferences and makes a decision consistent with them [5].

**Fig. 1:**
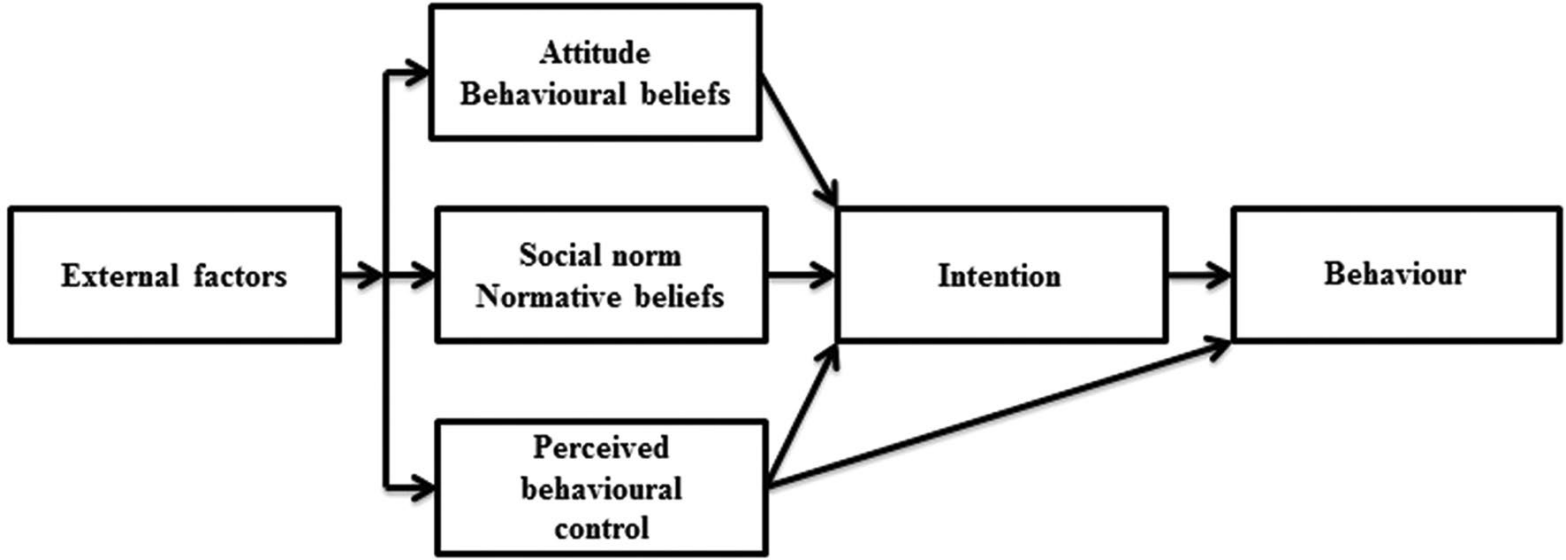
Theory of Planned Behavior framework

This significant paradigm shift encourages women to choose the option they feel most comfortable with [9–11]. The MHSS believes that women will wish to consult health professionals for assistance in making the decision, and that family physicians will be called upon to play this role. The MHSS therefore plans to develop interventions, including training programs, to better equip family physicians to this end. As intention has been repeatedly shown to predict behavior, we investigated whether family physicians intend to play this supporting role, and what might help or hinder the adoption of this behavior. The Theory of Planned Behavior (TPB) has proven an adequate measure of health professionals’ intention to adopt a new behavior (Fig. 1) [12–14]. The objectives of this study were therefore to use the TPB to measure family physicians’ intention to support women targeted by the QBCSP to make informed decisions about breast cancer screening with mammography, to identify determinants of this intention, and to identify barriers and facilitators that physicians perceive to adopting this behavior.

## Methods

For this cross-sectional survey we recruited a convenience sample of family physicians in the Province of Quebec who were attending one of two 45-min keynote presentations on informed decision making and cancer screening given by one of the co-authors (ML) during two major Continuing Medical Education conferences for family physicians in Montreal in 2010 (Additional file 1). After each lecture a 10-min questionnaire was distributed to all attendees who were invited to voluntarily complete the questionnaire anonymously before leaving the room. Return boxes were available at the door. No written consent was obtained from study participants. The project was exempted from ethical review by the Research Ethics Committee of Laval University, Quebec City, Canada.

### Data collection

The questionnaire, consisting of 13 questions in French, began with a brief introduction to the QBCSP, including its old and new slogans (Additional file 2). Questions 1–9 comprised 12 TPB-based items that measured respondents’ intention to support women targeted by the QBCSP in making informed decisions about breast cancer screening and assessed the determinants of this intention (attitude, socio-professional norm, and perceived behavioral control) [15]. Each of four socio-cognitive variables was assessed by means of three items, using a 6-point Likert scale ranging from −3 to +3. Questions associated with each of the four socio-cognitive variables are identified in the Additional file 2. The internal consistency of these variables was acceptable as depicted by their Cronbach’s alphas: intention (0.84); attitude (0.80); social norm (0.79); perceived behavioral control (0.77). Questions 10 and 11 collected socio-demographic data. Questions 12 and 13 were open-ended questions about factors that might hinder or facilitate family physicians in supporting women targeted by the QBCSP in making informed decisions about screening for breast cancer. A total of about 800 questionnaires were distributed during the two presentations, 350 in the first and 450 in the second.

### Data analysis

We excluded from the analysis family physicians who either did not answer any of the 12 items used to assess intention (three items) and its three determinants (three items each) or who answered only one of the three items used to assess each variable. We imputed missing values (Monte Carlo method) [16] for all respondents who answered two out of three items used in assessing each variable. The proportion of missing values was 126/3468 (3.6 %). We computed descriptive statistics for all variables collected. The proportion of family physicians with strong or very strong intention (score of 2 or more) and its 95 % confidence interval were calculated. Means are presented with their standard deviation.

**Fig. 2:**
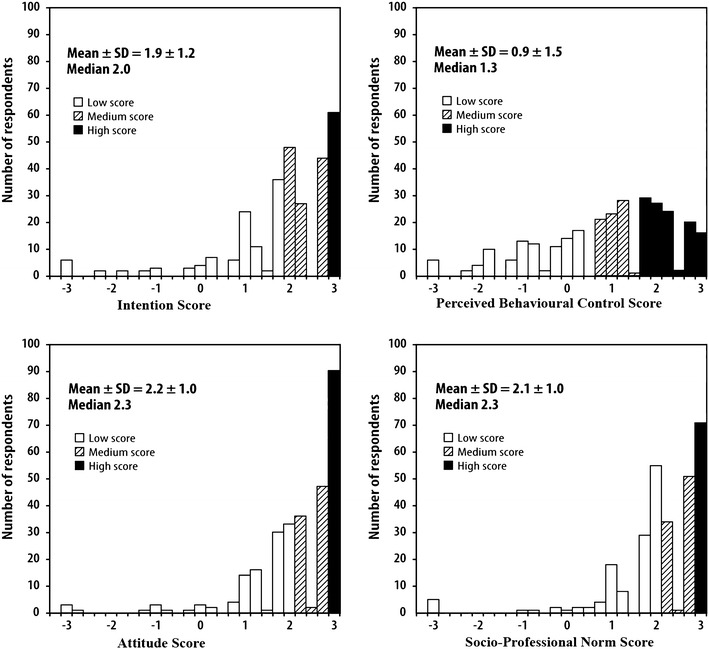
Scores of intention and its determinants to support women in making informed decisions about breast cancer screening

In order to identify which of the TPB determinants of intention might best explain the variation in intention, we calculated the Spearman’s rank correlation coefficient between intention and each variable. Then, to evaluate the adjusted effect of each variable on the intention, we performed a multivariate analysis. We used multinomial ordinal logistic regression instead of multiple linear regressions due to violation of the normality and linearity criteria, persisting even after attempting to transform the data. We divided scores of each variable into three categories according to terciles (Fig. 2). All analyses were done using the Statistical Analysis System software, Version 9.2 (SAS Institute, Cary, NC, USA).

Barriers and facilitators reported by family physicians were independently extracted and classified by ML, LNK, and a trained assistant using an adapted version of the taxonomy of shared decision making first developed by Légaré et al. [17]. and based on a framework by Cabana et al. [18]. According to this taxonomy, barriers and facilitators to supporting women in making informed decisions about breast cancer screening were classified into three main categories: (1) factors associated with knowledge of informed decision making concepts and techniques, (2) factors associated with attitude towards helping women in making informed decisions, and (3) external factors influencing behaviour (associated with patients, with shared/informed decision making as an innovation, and with environment) [17]. Discrepancies were resolved by consensus. The classification was done using Nvivo software Version 8.

### Availability of supporting data

All raw data are available from the authors upon request.

## Results

Out of the 350 questionnaires distributed at the first conference, 163 were returned, while 138 of the 450 questionnaires distributed in the second conference were returned, bringing the total estimated response rate to 38 %. The number of family physicians eligible for data analysis was 288, after excluding four who did not answer any of the 12 items used in assessing the variables as well as nine who answered only one out of the three items used in assessing each variable.

Participants were more likely to be female (63 %) than male (37 %). On average, male family physicians were more likely to have been longer in practice (26 ± 11 years) than their female counterparts (20 ± 11 years).

Figure 2 shows that distributions of the scores of all four variables were skewed to the left. Respondents had a strong intention to support women in making informed decisions about breast cancer screening: mean ± SD and median intention scores were 1.9 ± 1.2 and 2.0, respectively. The proportion of family physicians with strong or very strong intention (score of 2.0 or more) was 63 % (95 % CI 57–68 %).

There was a high correlation between intention and attitude (0.69, p < 0.001), social norm (0.64, p < 0.001), and perceived behavioral control (0.72, p < 0.001). Multinomial ordinal logistic regression analysis (Table 1) confirmed that all three determinants were independently associated with family physicians’ intention to support women targeted by the QBCSP in making informed decisions about screening for breast cancer, with a similar increment between low vs. medium/high intention score and low/medium vs. high intention score for each determinant. Only results from the model without missing values imputed are reported, as results with and without them were similar. The variable most strongly associated with intention was perceived behavioral control, followed by attitude, and then social norm. Gender and number of years in practice were associated neither with family physicians’ intention nor with any of the determinants of intention. The most frequently cited barrier was time constraints and the most frequently cited facilitator was availability of relevant information and decision support tools for both physicians and patients (Table 2).

**Table 1:** Association between family physicians’ intention and its determinants according to the Theory of Planned Behavior

Determinants of intention	Odds ratio^a^	95 % CI
Attitude		
High score	7.5	3.3–16.8
Medium score	3.8	1.9–7.6
Low score^b^	1	
Social norm		
High score	5.8	2.6–12.9
Medium score	1.7	0.9–3.3
Low score^b^	1	
Perceived behavioural control		
High score	15.7	6.7–36.8
Medium score	4.3	2.2–8.4
Low score^b^	1	

*CI* confidence interval

^a^Multinomial ordinal logistic regression model: Wald Chi Square for model = 124.9, *P* value <0.001; Score Test for the Proportional Odds Assumption, *P* value = 0.29 meaning that the odds ratios between low vs. medium/high intention score and low/medium vs. high intention score are assumed to be the same

^b^Low mean score: reference category

**Table 2:** Barriers and facilitators to supporting women in making informed decisions about breast cancer screening

Factors	Barriers	Facilitators
	n (%)	n (%)
Knowledge		
Knowledge about informed decision making	18 (6)	23 (8)
Attitude		
Self-efficacy	7 (2)	1 (0)
Motivation	8 (3)	4 (1)
External factors influencing behaviour		
Patients		
Characteristics of targeted women	18 (6)	–
Awareness of the relevant information among targeted women	7 (2)	26 (9)
Preference of targeted women	8 (3)	1 (0)
Environment		
Time	118 (41)	4 (1)
Relevant tools	10 (4)	84 (29)
Human resources	2 (1)	23 (8)
Remuneration	1 (0)	12 (4)
Organizational structure	8 (3)	4 (1)
Malpractice liability	4 (1)	1 (0)

*n (%)* number (proportion) of the 288 family physicians who cited a given factor

## Discussion

Family physicians in our study had strong intentions to support women in making informed decisions about breast cancer screening. Perceived behavioral control was the variable most strongly associated with their intention, followed by attitude and then social norm. These findings concur with the results of two systematic reviews assessing the efficacy of the TPB in predicting intention and behavior. They also found that health professionals perceived behavioral control as the strongest predictor of intention, followed by attitude, while social norm was the weakest predictor [12, 19]. However, ours is the first study using a theoretical model to assess family physicians’ intention to support women in making informed decisions about screening for cancer.

Although research findings illustrate the numerous benefits of informed and shared decision making (informed decisions by patients that involve both patient and practitioner) in clinical practice [6, 20, 21], health professionals still perceive many barriers to its implementation. In a systematic review on barriers and facilitators to implementing shared decision making in clinical practice [17], authors found that the three most reported barriers in 38 studies were time constraints (22/38), and lack of applicability due to patient characteristics (18/38) and clinical context (16/38). The three most reported facilitators were provider motivation (23/38), positive impact on the clinical process (16/38), and on patient outcomes (16/38). Family physicians in our study also identified time constraints, lack of relevant information and decision support tools as important barriers to supporting women in making informed decisions about breast cancer screening. It is unclear if using patient decision aids during consultation has a significant impact on the length of consultation. In a Cochrane review of nine randomized trials, the length of consultation in decision aids groups compared to usual care groups varied between −8 min and +23 min with a median of + 2.55 min; six trials reported no difference in the length of the consultation [21]. Physicians must however devote time to training to appropriately use shared/informed decision making techniques and tools.

Physicians also reported remuneration, organizational structure, self-efficacy, motivation, awareness, knowledge of the evidence, and the characteristics of the targeted women. According to the TPB, barriers and facilitators identified by physicians in our study are mainly associated with perceived behavioral control and attitude.

We also used a similar questionnaire with the same 12 TPB questions to survey (online) a sample of 840 primary care nurses in contact with women targeted by the QBCSP. Results of the survey demonstrated that perceived behavioral control was also the strongest predictor of nurses’ intention, followed by attitude. The main barriers limiting nurses’ support for women in making informed decision about breast cancer screening were lack of relevant decision support tools and training in informed decision making [22].

In response to the results of both studies, the MHSS in collaboration with l’Institut national de santé publique du Québec (National Public Health Institute of Quebec) and the Canada Research Chair in Implementation of Shared Decision Making in Primary Care at Université Laval created a 2-h theory-based online tutorial to help health professionals support women targeted by the QBCSP to make informed decisions about breast cancer screening with mammography. The tutorial focuses on shared/informed decision making techniques and tools emphasizing efficient use of consultation time. It is available (only in French) since 2013 at http://campusvirtuel.inspq.qc.ca/pages/decision-sein for a nominal fee and is provided free of charge since 2014 to all family physicians and nurses in the Province of Quebec at http://caducee.fmoq.org/ext/fmoq/accueilPublique.cnx.

### Strengths and limitations

This study demonstrated a number of strengths. Firstly, as intention has been repeatedly shown to predict behavior [14], we based our questionnaire on the Theory of Planned Behavior, a validated model that has been used in many international research projects to predict intention and its determinants vis-à-vis the adoption of healthcare behaviors [12]. Second, we complemented our assessment by asking physicians to identify barriers and facilitators to adopting the behavior, allowing us to make concrete suggestions for the design of interventions targeting behavior change. Third, according to systematic reviews, our study appears to be one of the largest assessments of health professionals’ intentions that use a socio-cognitive theory [12, 19].

This study also had some limitations. Our convenience sample of family physicians had just attended a presentation on informed/shared decision making and cancer screening, which might explain the high intention observed. Our results may not be generalizable to all family physicians in Canada or other countries and may be limited to physicians who just attended a similar presentation. In addition, the response rate in the selected sample was 38 %. This response rate may indirectly indicate lack of physicians’ interest in the topic and one could speculate that the intention of non-respondents would have been lower than in respondents. Nevertheless the response rate is high enough to indicate that a significant proportion of participants in the two CME events had a high intention to support women in making informed decisions about breast cancer screening. We do not know however if their intention was already strong or if it was a result of attending the presentation. Evaluating the effect of an intervention aimed at modifying the intention and behaviour of physicians would require collecting baseline data in participants and ideally in comparison with a control group.

Factors not identified in our study may constitute other barriers for supporting women in making informed decisions about breast cancer screening, for example, physicians’ misunderstanding of cancer screening statistics. Wegwarth et al. observed that 69 % of surveyed primary care physicians recommended a cancer screening test after being presented with irrelevant evidence—e.g. that finding more cases of cancer in screened as opposed to unscreened populations proves that screening saves lives—compared to 23 % who were presented with relevant evidence [23].

Finally, further research is needed to assess the actual behavior of family physicians in supporting women in making an informed decision about breast cancer screening. This might improve accurate targeting of interventions.

## Conclusion

Family physicians who participated in our study had strong intentions to support women targeted by the QBCSP in making an informed decision about breast cancer screening with mammography. Designing interventions that target perceived behavioral control, attitude, and social norm in family physician training and providing them and the targeted women with decision support tools should help women to make informed decisions about breast cancer screening.

## Additional files

10.1186/s13104-015-1608-8 Presentation on informed decision making and cancer screening (in French).
10.1186/s13104-015-1608-8 Questionnaire (translated from the original in French).
