# Supplementary material for: Family physicians’ intention to support women in making informed decisions about breast cancer screening with mammography: a cross-sectional survey
Source: BMC Res Notes. 2015 Nov 10;8:663. doi: 10.1186/s13104-015-1608-8 (PMC4640384; doi:10.1186/s13104-015-1608-8)
Supplement: Supplementary file 1 — 10.1186/s13104-015-1608-8 Presentation on informed decision making and cancer screening (in French). [file 13104_2015_1608_MOESM1_ESM.pdf]

Dépister ou ne pas dépister le cancer:

Comment décider avec son patient?

Michel Labrecque, MD, PhD

Département de médecine familiale et de médecine d'urgence

Université Laval

12 novembre 2010

UNIVERSITÉ LAVAL

Conflits d'intérêts potentiels

- Aucun

Avertissement

- Je vais vous donner une perspective différente de la question du dépistage du cancer.
- En insistant sur ce qui ne vous est pas familier, je vais paraître négliger ce qui l'est:
  le cancer est une maladie horrible.

Objectifs

À la fin de la présentation vous serez en mesure de:

- Juger de l'efficacité et des limites du dépistage des cancers de la prostate, du côlon et du rectum ainsi que du sein
- Transmettre les informations pertinentes à vos patients concernant le dépistage de ces cancers
- Vous engager dans un processus de décision partagée avec vos patients

Jean-Claude

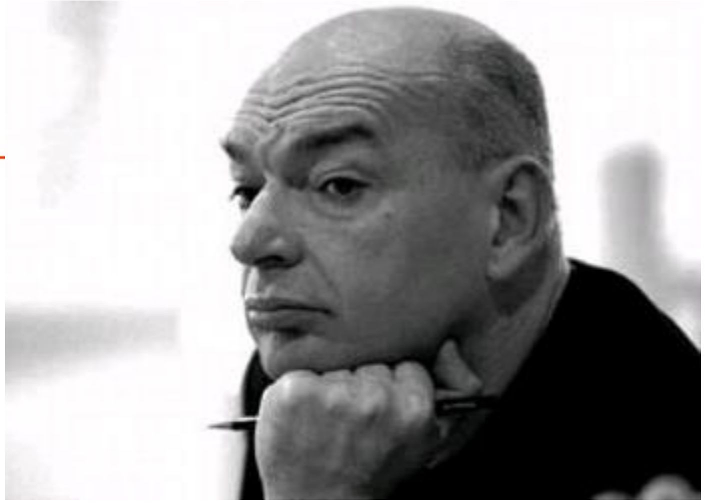

Jean-Claude, 60 ans, asymptomatique, sans antécédent personnel ou familial de cancer de la prostate, désire « bilan de santé pour sa prostate ». Son voisin et ami qui a 62 ans vient tout juste d'apprendre qu'il a un cancer de la prostate.

Que conseillez-vous à Jean-Claude?

Marie

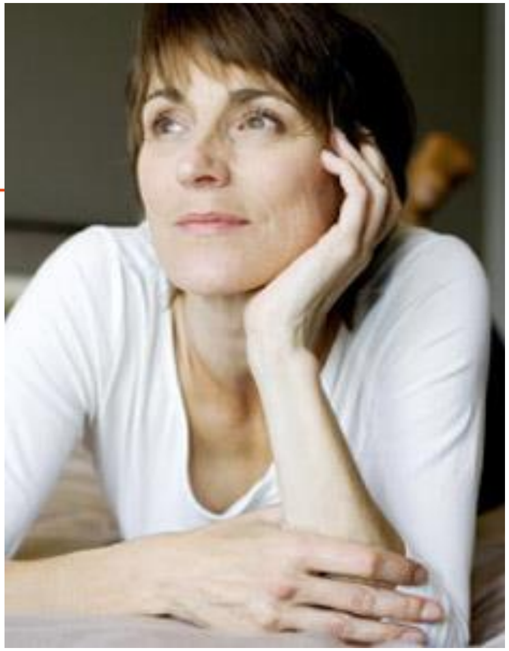

Marie, 50 ans, asymptomatique, sans antécédent personnel ou familial de cancer du sein a reçu l'invitation à participer au Programme québécois de dépistage du cancer du sein (PQDCS). Elle ne sait trop quoi penser suite à la lecture des documents d'information qui accompagnait la lettre d'invitation. Elle désire votre avis.

Que conseillez-vous à Marie?

Jean-Claude et Marie

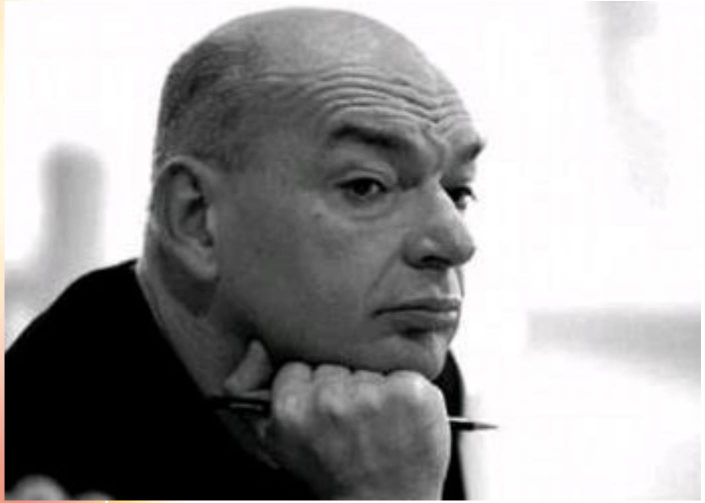

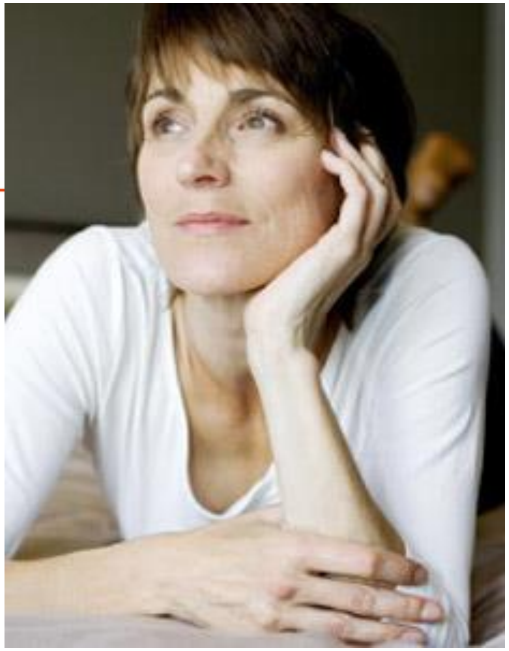

…et docteur, pour le cancer des intestins?

UNIVERSITÉ LAVAL

Que conseillez-vous à Marie et Jean Claude?

Sauver votre vie grâce au dépistage!

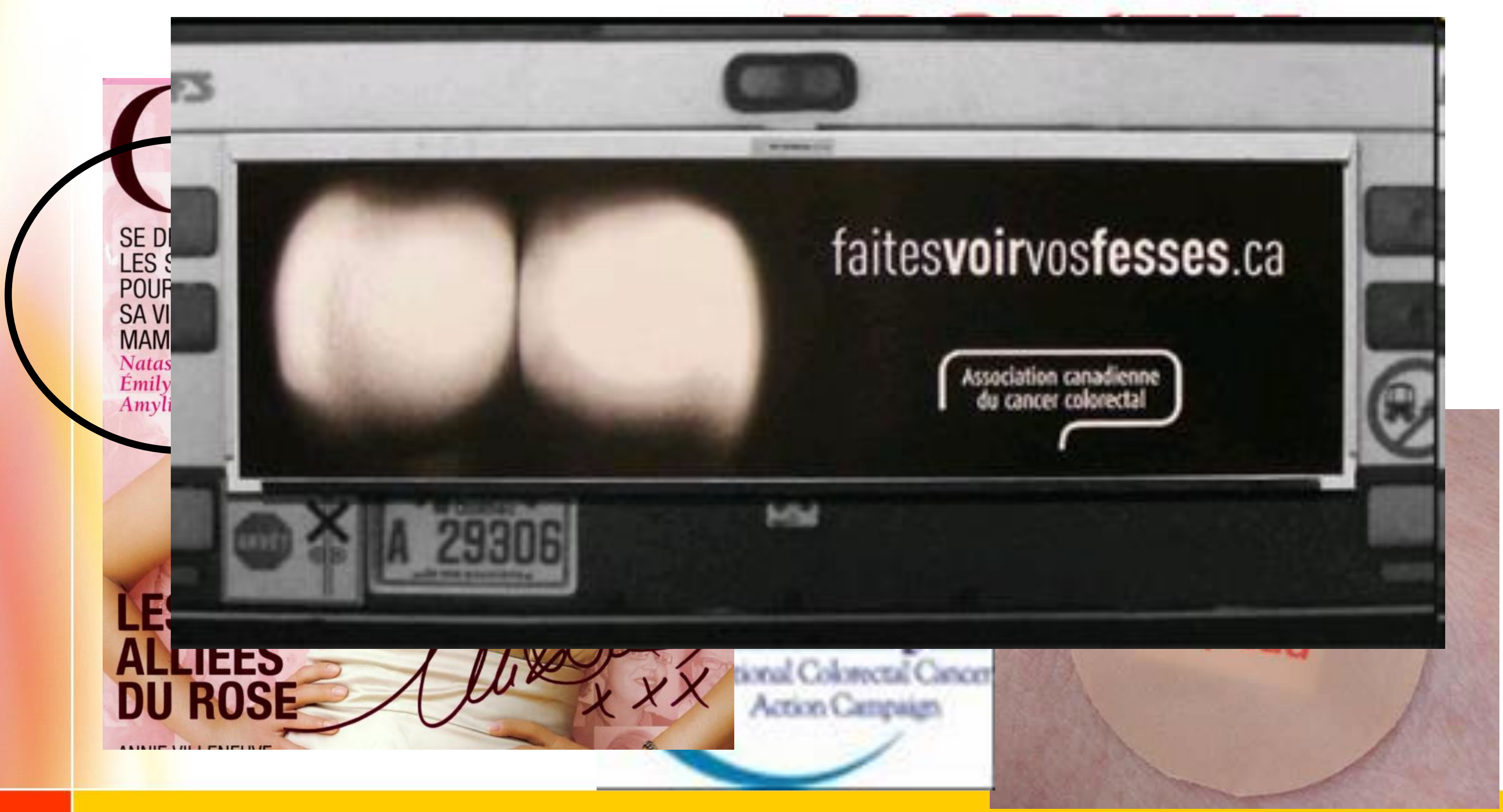

UNIVERSITÉ LAVAL

Sauver votre vie grâce au dépistage!

Gouvernement

Publications scientifiques

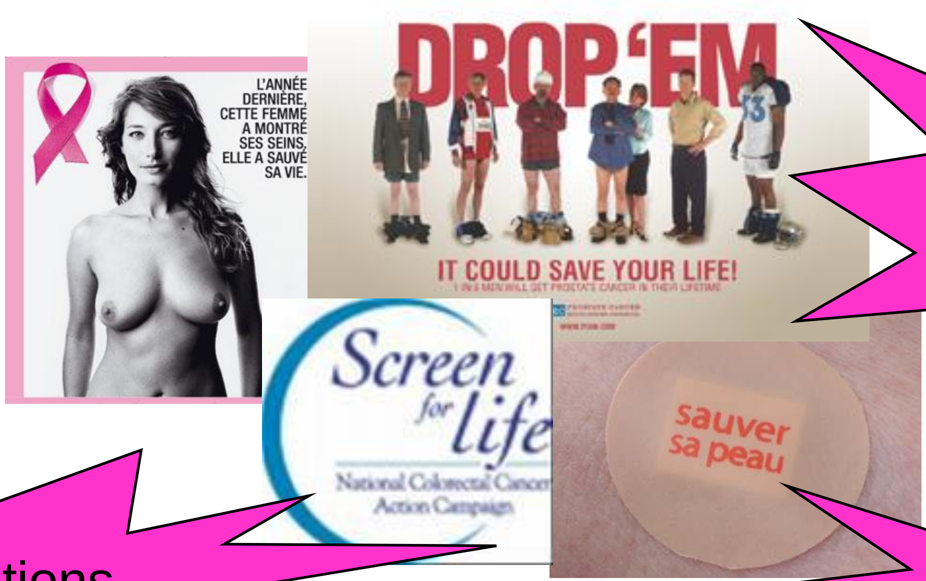

Associations de « survivants»

Recommandations d'experts

Associations professionnelles

Sauver votre vie grâce au dépistage!

Gouvernement

Bien faire notre travail

Publications scientifiques

Peur de manquer un cancer

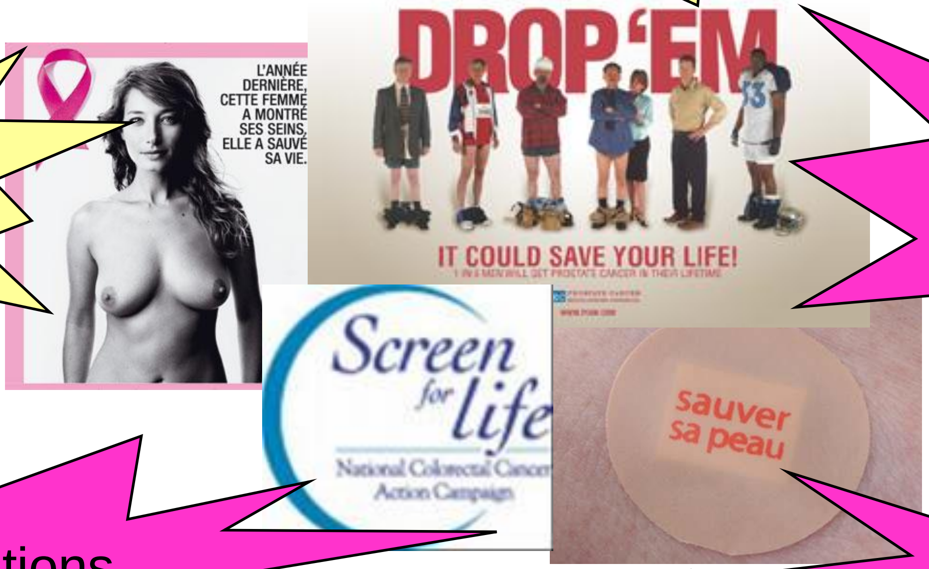

Associations de « survivants»

Recommandations d'experts

Demande de nos patients

Associations professionnelles

UNIVERSITÉ LAVAL

Rendre « malade » une personne « en santé » en assumant que, grâce à nos interventions, elle sera en meilleure santé à plus long terme.

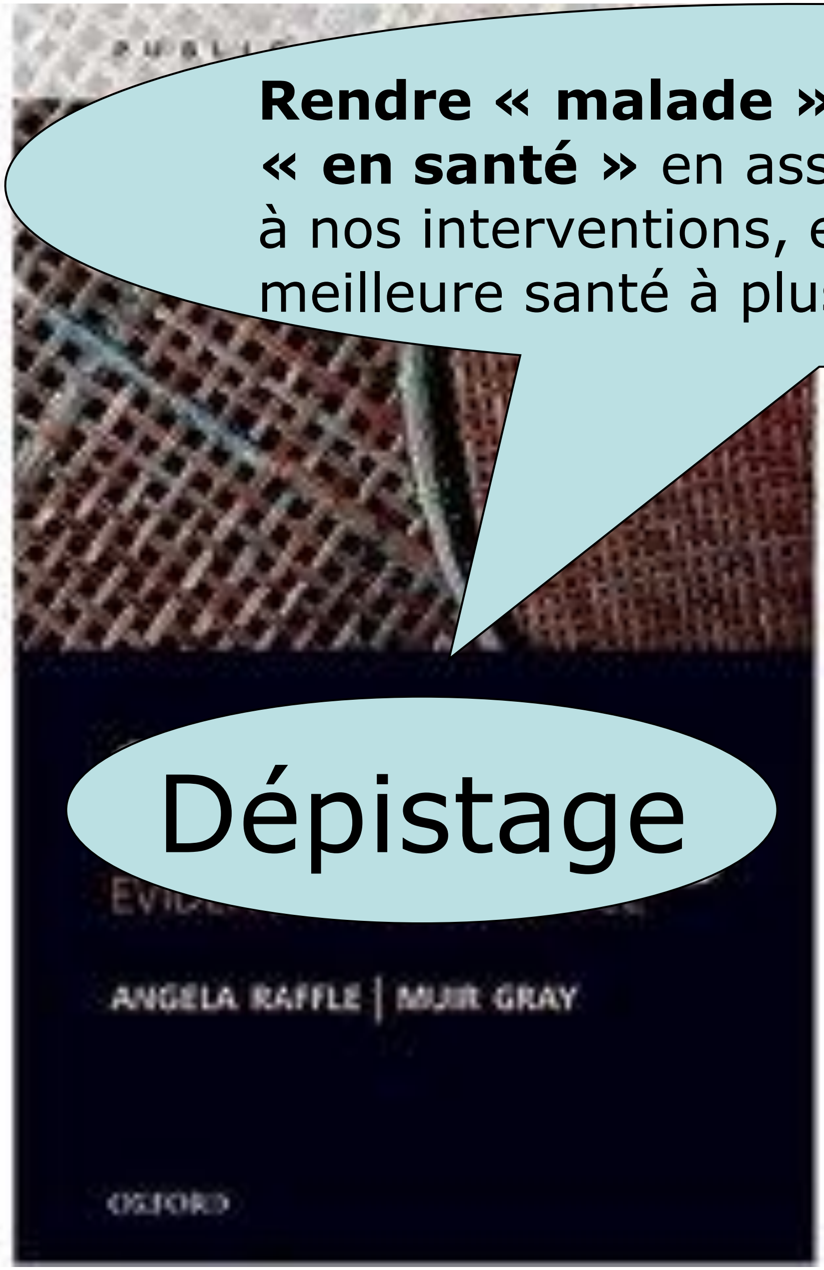

Dépistage

Tous les programmes de dépistage font du tort. Certains font également du bien, et parmi ces derniers, certains le font à un coût raisonnable...

Raffle & Gray 2007

Le dépistage du cancer est une loterie!

- De rares grands gagnants
  - dont la vie est allongée
- Quelques grands perdants
  - rassurés à tort
  - traités inutilement
- Plusieurs petits perdants
  - Étiquetés à tort comme malades
- Une multitude de petits gagnants
  - Rassurés à juste titre

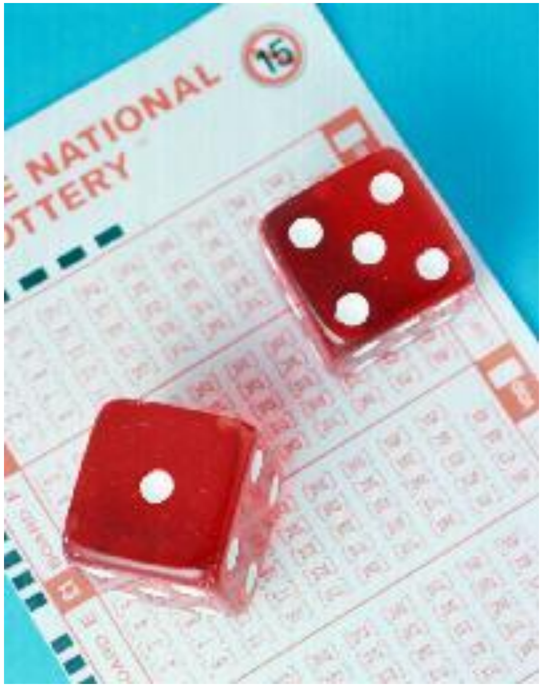

Sans pouvoir les distinguer a priori!

UNIVERSITÉ LAVAL

Qu'est ce que la prise de décision partagée?

Une processus au cours duquel le professionnel de la santé et le patient **prennent ensemble une décision** fondée sur les **meilleures données scientifiques** disponibles sur les **bénéfices et les risques** de toutes les options tout en considérant les **valeurs et préférences** du patient à cet égard.

Intégration de la Médecine fondée sur les données probantes et l'Approche centrée sur le patient

UNIVERSITÉ LAVAL

Une décision de qualité

Congruence

Choix

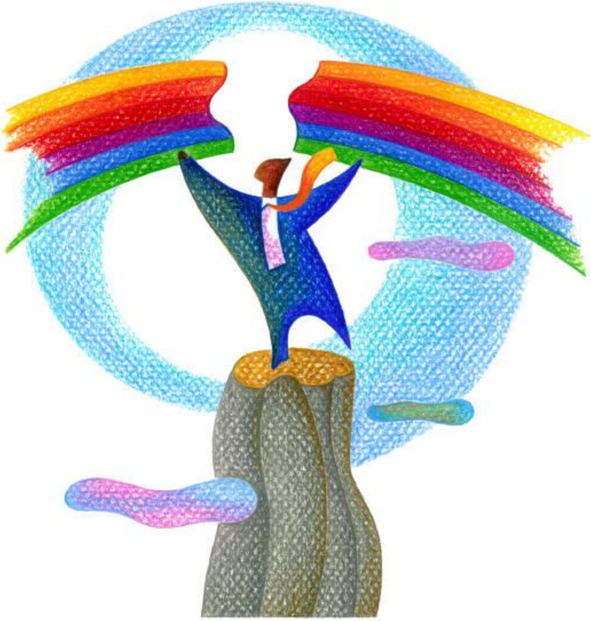

Ce que le patient informé valorise

International Patient Decision Aids Standards 2006

Et puis après…

Comment je fais ça?

Ai-je temps ?

Est-ce la “bonne” patiente?

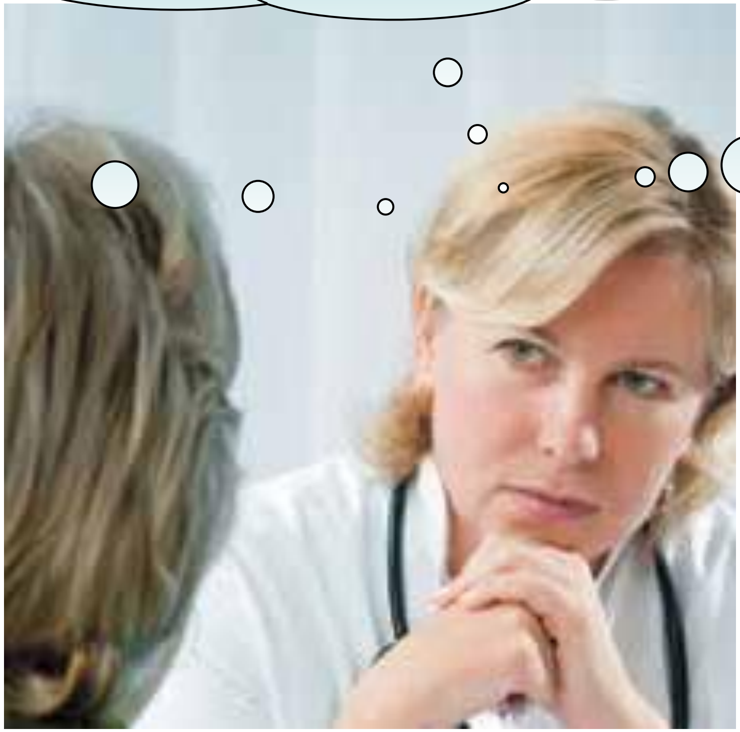

UNIVERSITÉ LAVAL

Légaré et al. Patient Education and Counselling 2008

Les 4 étapes pour intégrer la prise de décision partagée en pratique

1. Initier un processus de prise de décision partagée
2. Partager de façon claire et balancée les informations pertinentes
3. Favoriser la participation active du patient à la prise de décision
4. S'assurer que le patient est confortable avec sa décision

Initier un processus de prise de décision partagée

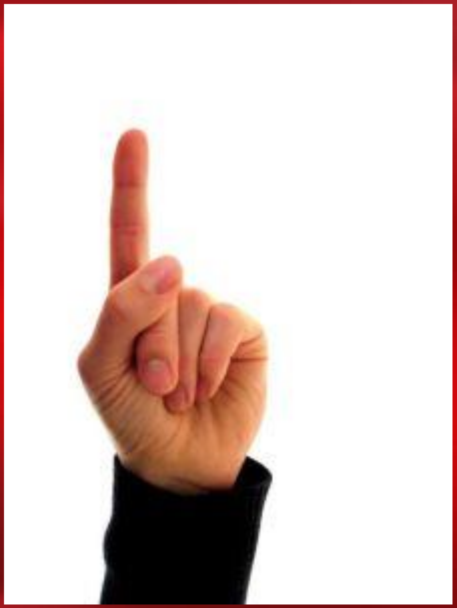

UNIVERSITÉ LAVAL

Initier un processus de prise de décision partagée

- Expliquer à votre patient qu'il y a une décision à prendre (dépister ou ne pas dépister).
- Préciser qu'il n'y a pas un bonne ou un mauvaise option, seulement une option avec laquelle on est plus confortable. Les deux options sont acceptables.
- Vérifier le désir du patient et son confort à participer à la prise de décision.

UNIVERSITÉ LAVAL

Jean-Claude, nous devons prendre un décision concernant le dépistage du cancer de la prostate. Certains hommes choisissent de le faire alors que d'autres hommes décident de ne pas le faire. Les deux sont acceptables, mais il y a des choses que vous devez savoir avant de décider…

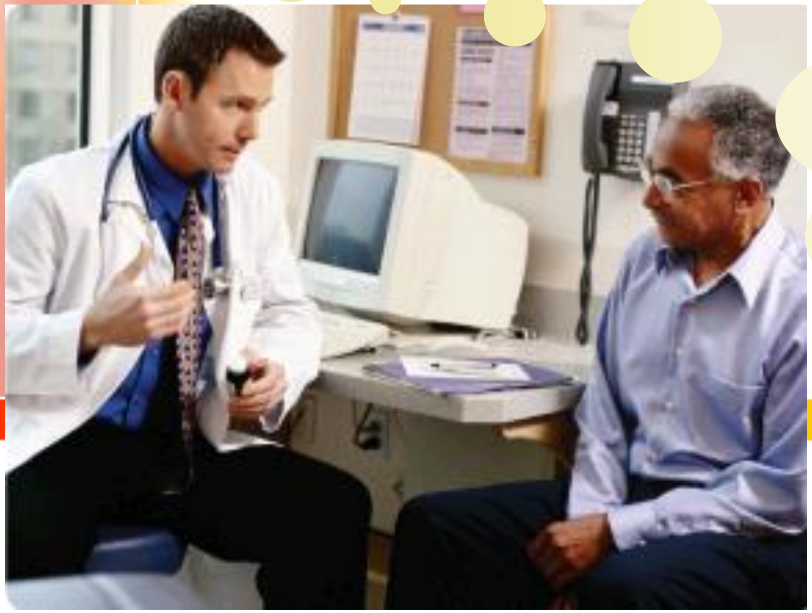

... nous pourrons ensuite décider ensemble selon ce que ce vous jugerez être le mieux pour vous. Est-ce que ça vous convient?

Partager de façon claire et balancée les informations pertinentes

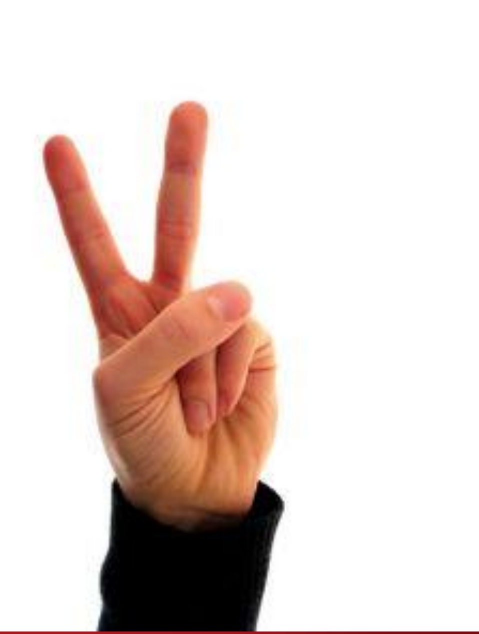

UNIVERSITÉ LAVAL

Informations à partager

- Risque d'avoir *vs* de mourir du cancer
- Test de dépistage
- Bénéfices potentiels
  - allonger sa vie (vrai +)
  - être rassuré de ne pas avoir de cancer (vrai -)
- Risques potentiels
  - être rassuré à tort (faux –)
  - être inquiété à tort (faux +)
  - être très inquiété et traité à tort (« faux » vrai +)
- Caractère probabiliste et incertitude scientifique

Et puis après…

Est-ce que je connais les données?

Les données sont-elles sont valides?

Comment lui expliquer tout ça?

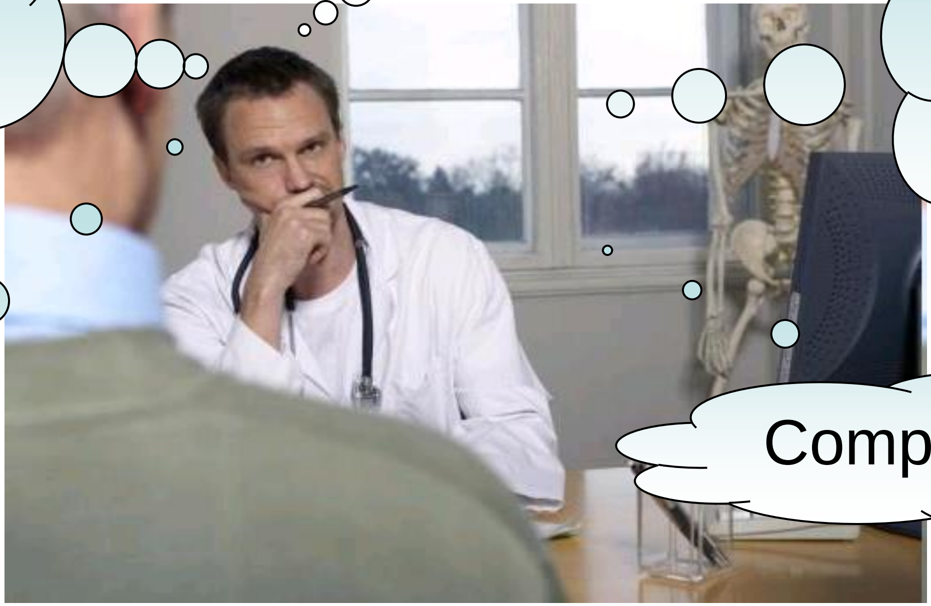

Ai-je temps ?

Comprendra-t-il?

Légaré et al. Patient Education and Counselling 2008

Cancer de la prostate

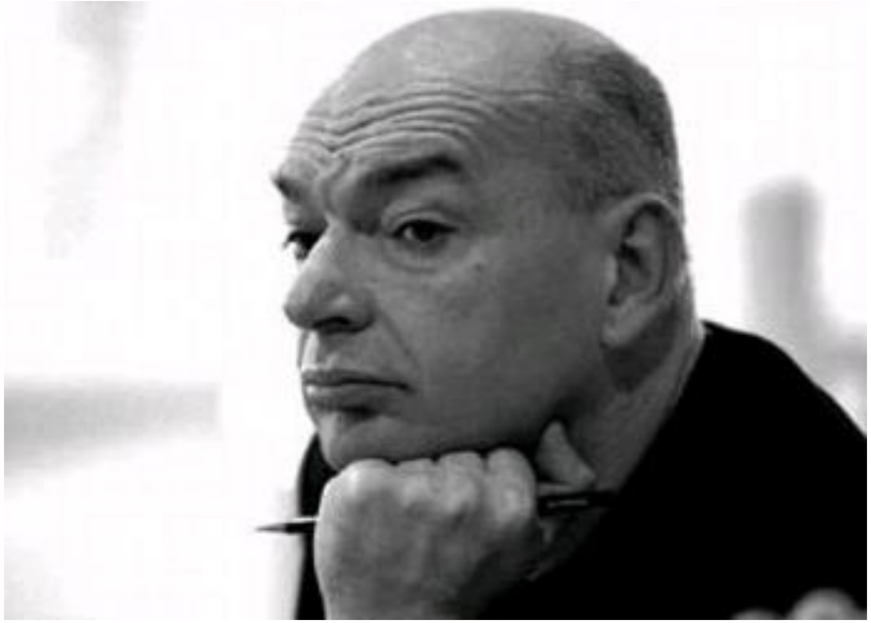

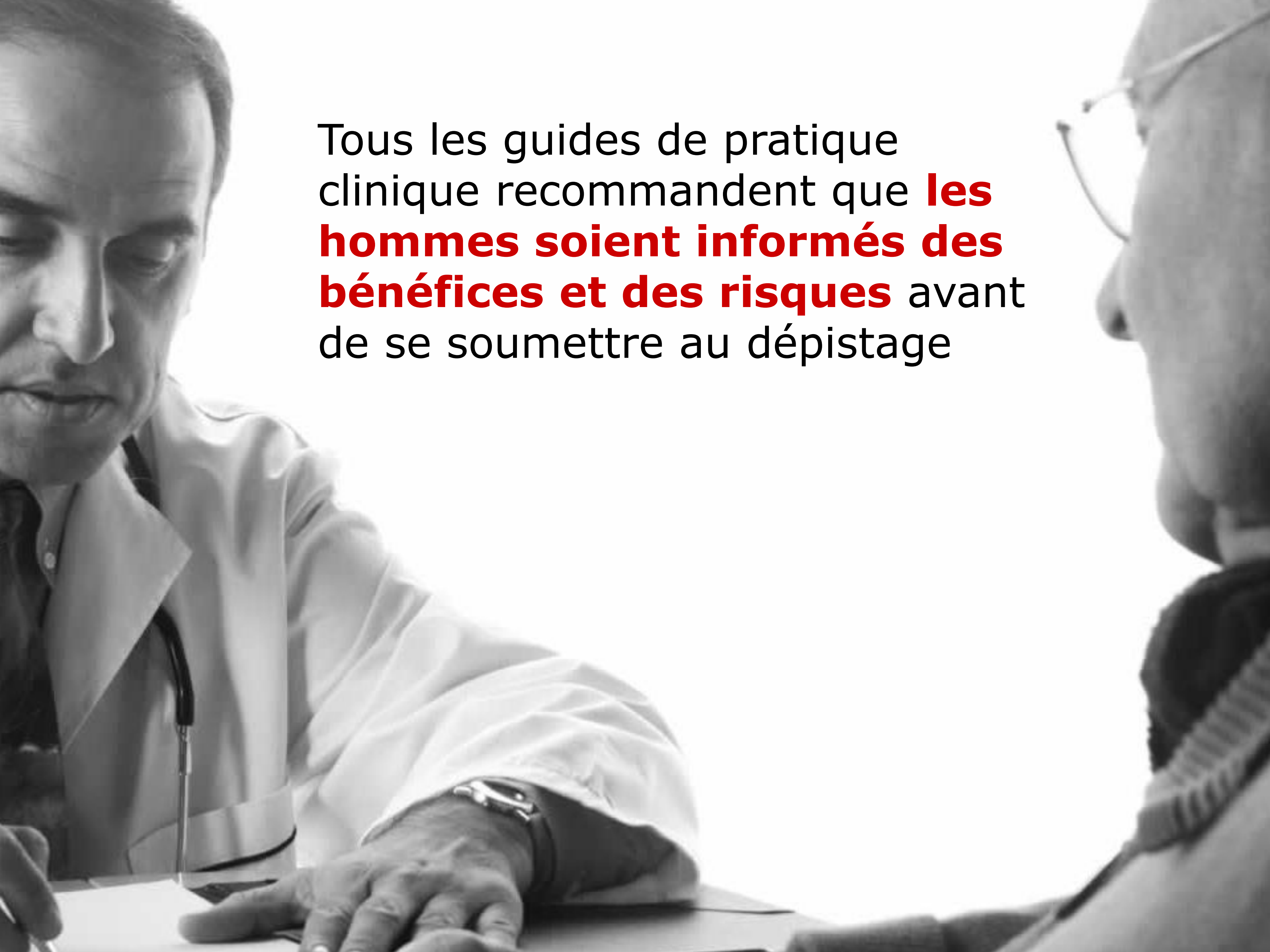

Tous les guides de pratique clinique recommandent que **les hommes soient informés des bénéfices et des risques** avant de se soumettre au dépistage

Vivre plus longtemps grâce au dépistage ?

1000 hommes de 60 ans sans antécédents familiaux de cancer de la prostate ont un dosage sérique d'**APS** annuellement pendant **10 ans**. Combien ont leur vie allongée au-delà de ces 10 ans grâce au dépistage?

- [ ] Aucun
- [x] 1
- [ ] 10
- [ ] 38
- [ ] 122

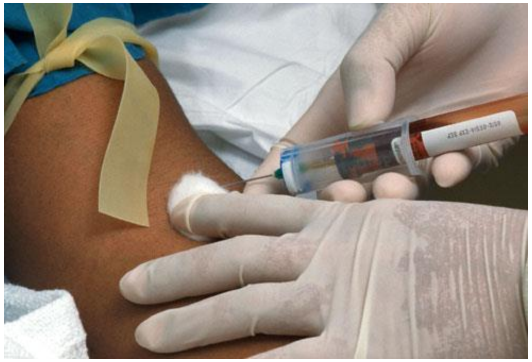

UNIVERSITÉ LAVAL

Howard et al 2009

Résultat « net » du dépistage annuel du cancer de la prostate pendant 10 ans chez 1000 hommes de 60 ans sans antécédent familial

(+)

- 1 a sa vie allongée (?)
  - 2 à vie (?)
- Être rassuré (?)
  - 850-900 dépistage -
  - Mais 3 à 20+ cancers manqués

(-)

- Être inquiété à tort
  - 100-150 biopsies de plus
    - 75-100 sans cancer
    - 10-15 avec complications
- Être traité à tort
  - 30 Dx et Rx de plus (?)
    - 15 avec complications

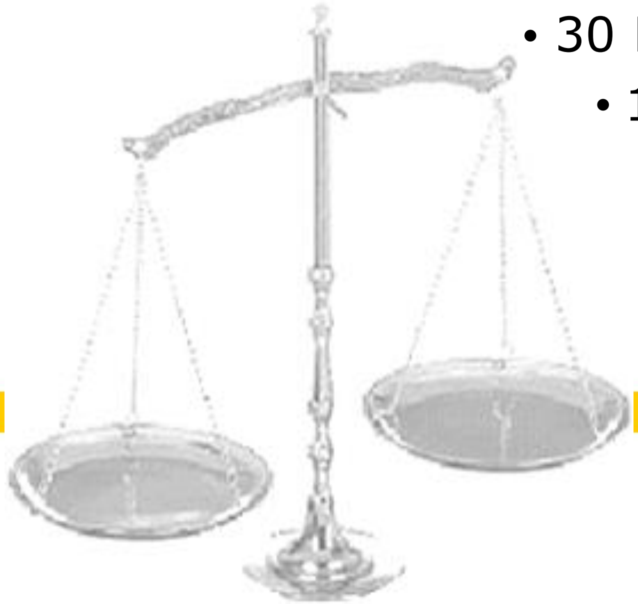

UNIVERSITÉ LAVAL

Howard et al 2009

Résultat « net » du dépistage annuel du cancer de la prostate pendant 10 ans chez 1000 hommes de 60 ans sans antécédent familial

(+)

- 1 a sa vie allongée (?)
  - 2 à vie (?)
- Être rassuré (?)
  - 850-900 dépistage -
  - Mais 3 à 20+ cancers manqués

(-)

- Être inquiété à tort
  - 100-150 biopsies de plus
    - 75-100 sans cancer
    - 10-15 avec complications
- Être traité à tort
  - 30 Dx et Rx de plus (?)
    - 15 avec complications

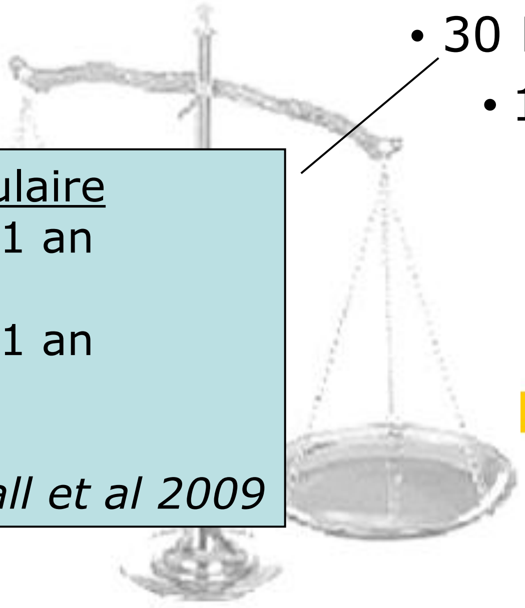

<u>Risque de décès cardio-vasculaire</u>
RR 2,8 à 1 semaine et 1,3 à 1 an
<u>Risque de suicide</u>
RR 8,4 à 1 semaine et 2,6 à 1 an

Plus élevé < 54 ans

Fall et al 2009

Howard et al 2009

es plus visités Débuter avec Firefox À la une Dr Michel Labrecque

Outil d'aide à la décision pour le dépi...

Accueil Se connecter

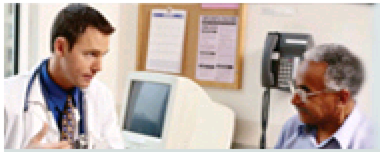

Outil d'aide à la décision
pour le dépistage du cancer de la prostate

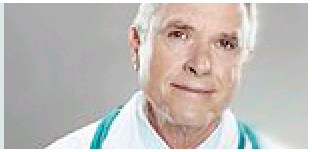

- Le cancer de la prostate
- Le dépistage
- Évaluer votre risque
- Une décision à prendre
- Autres sources d'information
- Quelle est votre décision?
- Qui sommes-nous?
- Avis de non-responsabilité

Accueil

BIENVENUE DANS CET OUTIL D'AIDE À LA DÉCISION POUR LE DÉPISTAGE DU CANCER DE LA PROSTATE.

Ce site a été conçu pour vous informer et vous aider à décider de faire ou non le dépistage du cancer de la prostate.

Il s'adresse aux hommes de 40 à 50 ans avec une histoire de cancer de la prostate dans la famille proche et aux hommes de 50 à 69 ans avec ou sans histoire de cancer de la prostate dans la famille. Chez les autres hommes, dont les hommes plus âgés, la majorité des organismes en santé ne recommandent pas de faire le dépistage du cancer de la prostate. Les mêmes organismes ne recommandent également pas de faire le dépistage chez les hommes chez qui on estime qu'ils auraient moins de 10 années à vivre à cause d'autres problèmes de santé.

© Droits réservés, Université Laval 2010

infoprostate.fmed.ulaval.ca

né zot

es plus visités Débuter avec Firefox À la une Dr Michel Labrecque
Outil d'aide à la décision pour le dépi...

Outil d'aide à la décision pour le dépistage du cancer de la prostate

Les avantages et les inconvénients du dépistage

Les chiffres qui vous seront présentés donnent une idée de ce qui arrive à 1000 hommes de votre âge et qui ont la même histoire familiale que vous et qui se font dépistés chaque année pendant 10 ans avec l'APS comparativement à ceux qui ne se font pas dépistés. Les chiffres sont fondés sur les données scientifiques les plus fiables et les plus optimistes à ce jour.[1,2] Il faut savoir qu'ils pourraient changer (en pire ou en mieux) quand les résultats d'autres recherches qui sont actuellement en cours seront disponibles.

1. *Howard K, Barratt A, Mann GJ, Patel MI. A model of prostate-specific antigen screening outcomes for low- to high-risk men: information to support informed choices. Arch Intern Med 2009;169:1603-10.*
2. *2. Hugosson J, Carlsson S, Aus G, et al. Mortality results from the Göteborg randomised population-based prostate-cancer screening trial. Lancet Oncol 2010;11:725-32.*

Veuillez maintenant répondre aux deux questions suivantes:

1. Quel âge avez-vous? 60

2. À votre connaissance, combien de membres de votre famille immédiate (votre père, un de vos frères ou un de vos enfants) ont eu un cancer de la prostate :

(●) Aucun ( ) 1 ( ) 2 ou plus

Valider

Voici les informations dont vous avez besoin pour prendre votre décision:

Avantages d'un dépistage annuel avec l'APS pendant 10 ans

né zot

es plus visités Débuter avec Firefox À la une Dr Michel Labrecque
Outil d'aide à la décision pour le dépi...

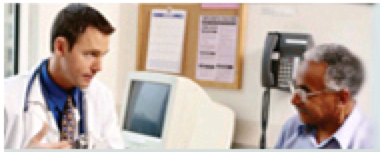

Outil d'aide à la décision
pour le dépistage du cancer de la prostate

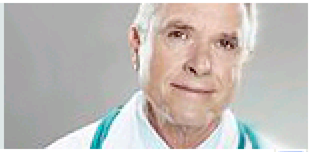

Avantages d'un dépistage annuel avec l'APS pendant 10 ans

Vous pouvez avoir un cancer de la prostate trouvé à un stade plus précoce et le faire traiter à ce stade.

Ainsi chez 1000 hommes de 60 ans qui optent pour le dépistage, pendant 10 ans, on estime que **53** ont un diagnostic de cancer de la prostate. Chez 1000 hommes de 60 ans qui ne font pas le dépistage, **23** ont un cancer détecté, parce que ces cancers ont donné des symptômes, habituellement des problèmes urinaires. C'est donc **30** hommes de plus dont le cancer est détecté, la majorité à un stade précoce.

Vous pouvez éviter de mourir du cancer de la prostate.

Trouver plus de cancers n'est pas le but du dépistage. Participer au dépistage doit pouvoir réduire le risque de mourir du cancer de la prostate et allonger la vie. Voici les estimations les plus optimistes:

Au cours des 10 prochaines années, on estime que sur 1000 hommes semblables à vous, il y en a **1** de moins qui meurent du cancer de la prostate à cause du dépistage: **3** meurent du cancer de la prostate parmi ceux qui font le dépistage et **4** parmi ceux qui ne font pas le dépistage.

On calcule aussi qu'avant l'âge de 85 ans, il y en a **2** de moins qui meurent du cancer de la prostate à cause du dépistage: **29** meurent du cancer de la prostate parmi ceux qui font le dépistage et **31** parmi ceux qui ne font pas le dépistage.

Il faut savoir cependant qu'on ne sait pas si globalement le dépistage permet d'allonger la durée de la vie, car la majorité des hommes meurent d'autres causes que le cancer de la prostate.

Inconvénients d'un dépistage annuel avec l'APS pendant 10 ans

Vous pouvez être rassuré qu'il n'y a pas de cancer alors qu'il y en a un.

Un résultat négatif aux tests ne garantit par l'absence de cancer. On a vu que sur les 10 ans, parmi les 1000 hommes qui optent pour le dépistage, **53** auront un diagnostic de cancer de la prostate. Cependant, **28** sont découverts par le dépistage fait à chaque année, alors que **25** le sont à cause de problèmes de santé survenant soit entre les dépistages (**3**) ou soit, comme cela arrive fréquemment, chez des

né zot

es plus visités Débuter avec Firefox À la une Dr Michel Labrecque

Outil d'aide à la décision pour le dépi... +

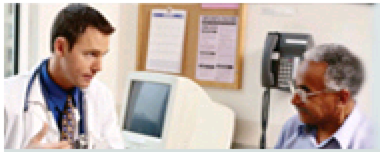

Outil d'aide à la décision
pour le dépistage du cancer de la prostate

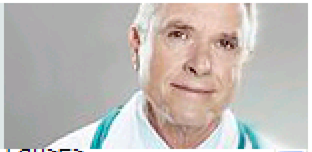

d'allonger la durée de la vie, car la majorité des hommes meurent d'autres causes que le cancer de la prostate.

Inconvénients d'un dépistage annuel avec l'APS pendant 10 ans

Vous pouvez être rassuré qu'il n'y a pas de cancer alors qu'il y en a un.

Un résultat négatif aux tests ne garantit par l'absence de cancer. On a vu que sur les 10 ans, parmi les 1000 hommes qui optent pour le dépistage, **53** auront un diagnostic de cancer de la prostate. Cependant, **28** sont découverts par le dépistage fait à chaque année, alors que **25** le sont à cause de problèmes de santé survenant soit entre les dépistages (**3**) ou soit, comme cela arrive fréquemment, chez des hommes qui ont eu un dépistage ou un suivi irrégulier (**22**).

Vous pouvez être inquiété d'avoir un cancer alors qu'il n'y en a pas.

La plupart des hommes dont le résultat au dépistage est positif n'ont pas le cancer de la prostate. Cependant, le résultat peut entraîner des effets psychologiques négatifs chez certains hommes. Pour savoir s'il y a un cancer ou non, on demande de faire des biopsies de la prostate à l'aide d'une sonde que l'on passe par le rectum.

On estime que chez 1000 hommes semblables à vous, le dépistage demande de faire **115** biopsies de plus sur les 10 ans et **87** de ces biopsies ne montrent pas de cancer. Des complications comme du sang dans les urines et une infection surviennent dans **12** (10%) de ces biopsies.

Vous pouvez être traité pour un cancer qui ne vous aurait jamais donné de problèmes.

La majorité des hommes qui sont traités pour un cancer trouvé par dépistage meurent d'autres causes que le cancer de la prostate. Il est difficile de savoir exactement combien d'hommes sont traité inutilement, mais c'est probablement la majorité. On estime que, parmi 1000 hommes qui participent au dépistage, il faut traiter par chirurgie entre 15 et 50 hommes qui ont un cancer de la prostate pour que 1 ait sa vie allongée au-delà de 10 à 15 ans.

Il faut savoir que tous les hommes qui se font enlever la prostate par chirurgie courent des risques d'avoir des complications du traitement. Environ entre 8 et 25 hommes (la moitié) souffrent d'impuissance et entre 2 et 5 (10%) d'incontinence des urines.

né zot

es plus visités Débuter avec Firefox À la une Dr Michel Labrecque

Outil d'aide à la décision pour le dépi... +

Accueil Se connecter

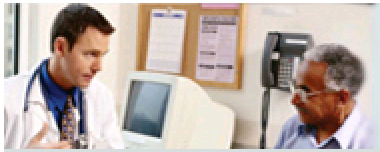

Outil d'aide à la décision
pour le dépistage du cancer de la prostate

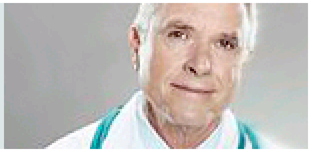

- Le cancer de la prostate
- Le dépistage
- Évaluer votre risque
- Une décision à prendre
- Autres sources d'information
- Quelle est votre décision?
- Qui sommes-nous?
- Avis de non-responsabilité

Accueil > Autres sources d'information

a⁻ A⁺

Autres sources d'information

Voici quelques sources d'information supplémentaires pour aider à prendre une décision concernant le dépistage du cancer de la prostate:

1) une boîte à décision qui résument les bénéfices et les risques que l'on a observé en moyenne avec le dépistage (2010)
2) un article qui s'intitule Le dépistage du cancer de la prostate. Une loterie! (2009)
3) un feuillet d'information du Collège des médecins du Québec (1998)
4) un feuillet d'information fondé sur les renseignements prodigués par le Programmes de dépistage du cancer du NHS en Grande-Bretagne (2009)
5) une vidéo (en anglais) présentant une entrevue avec le Dr. Otis Brawley, médecin responsable de l'American Cancer Society (2010)

Pour poursuivre, cliquez sur **Quelle est votre décision?** dans le menu de gauche.

infoprostate.fmed.ulaval.ca

né zot

BOÎTE À DÉCISION: DÉPISTAGE DU CANCER DE LA PROSTATE
par dosage de l'antigène prostatique spécifique (APS)

**À quoi sert ce test?**	Le dosage de l'APS permet de détecter le cancer de la prostate à un stade plus précoce et de le traiter à ce stade.
**Ce test est-il fiable?**	Le test peut parfois manquer un cancer et rassurer à tort ou bien faire suspecter un cancer alors que le plus souvent il n'y en a pas. Il peut aussi détecter un cancer à évolution lente ne s'accompagnant d'aucun symptôme et mener à des traitements inutiles.
**Qui pourrait considérer l'utiliser?**	Les hommes de 50 ans et plus, les hommes de 40 ans ou plus ayant un père ou un frère atteint du cancer de la prostate, ou les hommes de race noire.
**Pourquoi y a-t-il une décision à prendre?**	Le dépistage peut être bénéfique pour certains hommes alors qu'il peut causer du tort à d'autres

RÉSULTATS DES ÉTUDES SUR LE DÉPISTAGE DU CANCER DE LA PROSTATE *

Qu'arrive-t-il à des hommes entre 55 et 69 ans qui font le dépistage chaque année pendant 10 ans ?

Bénéfices du dépistage

Environ 1 décès dû au cancer de la prostate pourra être évité chez 1000 hommes qui font le dépistage.

Si ces hommes étaient suivis plus de 10 ans, alors quelques décès de plus pourraient probablement être évités.

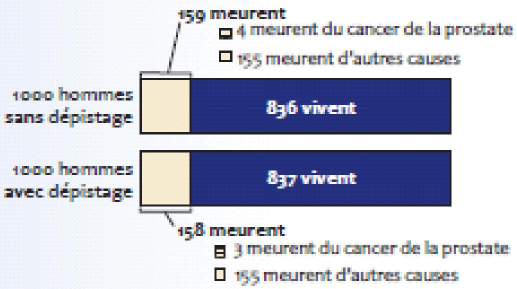

Risques du dépistage

Environ 140 hommes de plus sur les 1000 subiront une biopsie – la majorité apprendront qu'ils n'ont pas de cancer de la prostate.

Ainsi, plus d'hommes souffriront de complications dues à la biopsie:

- environ 2 hommes de plus seront hospitalisés.
- environ 4 hommes de plus auront une infection devant être traitée avec des antibiotiques.

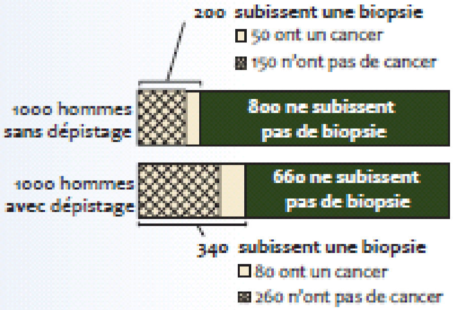

Environ 30 hommes de plus seront traités pour un cancer de la prostate. Chez la majorité, ce cancer n'aurait jamais affecté leur santé.

Ainsi, plus d'hommes auront à vivre avec des complications dûes aux traitements:

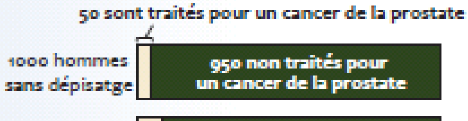

Cancer colorectal

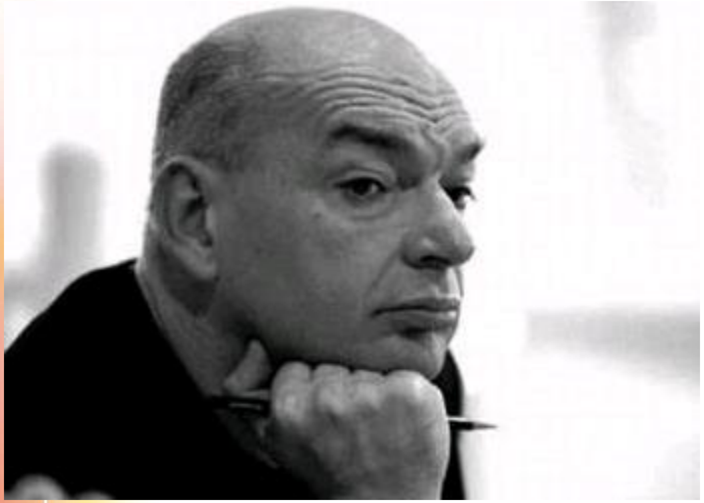

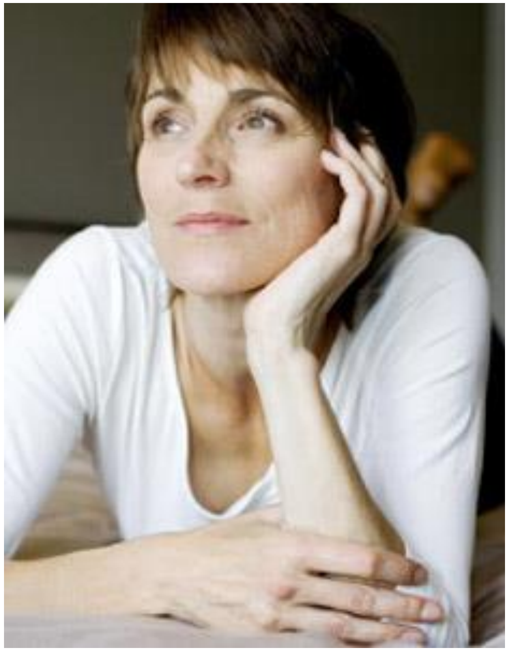

Les recommandations des Groupes d'étude sur les soins de santé préventifs

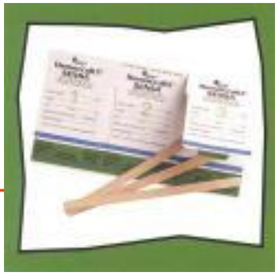

<u>Canada (2001)</u>

- 50+ ans
  - Recherche de sang occulte dans les selles (RSOS) (A)
  - sigmoïdoscopie (B)

<u>États-Unis (2008)</u>

- 50-75 ans (A)
  - RSOS
  - sigmoïdoscopie
  - coloscopie

Vivre plus longtemps grâce au dépistage ?

1000 individus de **45-80 ans** ont été invités à faire le dépistage du cancer colorectal par **recherche de sang occulte dans leurs selles aux 2 ans**. Combien ont eu leur vie allongée au-delà de 12-13 ans grâce à ce programme?

- ☑ Aucun
- ☐ 2
- ☐ 15
- ☐ 34
- ☐ 112

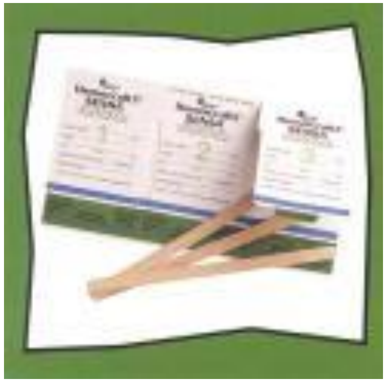

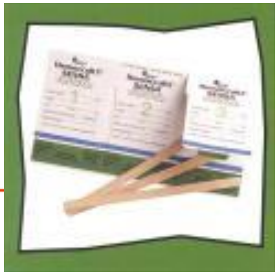

Dépistage du cancer colo-rectal: des avantages certains?

Mortalité chez 1000 individus de 45-80 ans invités à participer ou non au dépistage par RSOS chaque 2 ans pendant 12-13 ans.

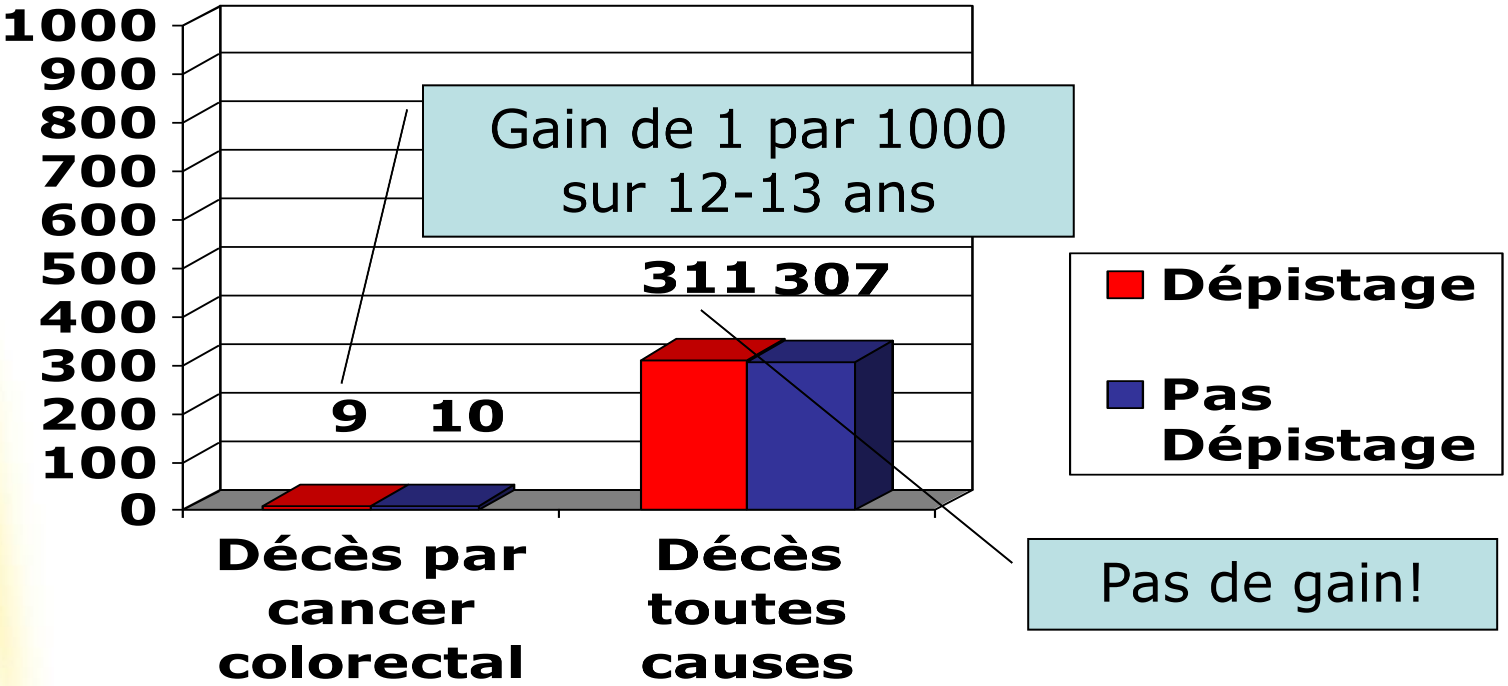

Cochrane 2006

Dépistage du cancer colorectal: des avantages certains?

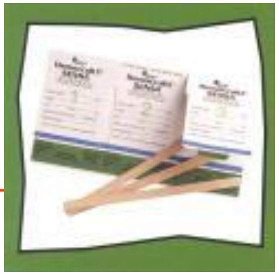

- «La recherche de sang occulte dans les selles aux 2 ans réduit la mortalité par cancer colorectal de 14% après 10 ans **sans preuve de bénéfices après cette période**. La **mortalité totale n'est pas modifiée** par cette stratégie de dépistage et des programmes s'appuyant sur cette mesure de dépistage ne seraient pas justifiés!»
  - *Heresbach et al. 2006*

- «**L'impact** du dépistage du cancer colorectal par la RSOS **sur la mortalité totale est incertain** et l'efficacité de cette intervention préventive demande à être réévaluée.»
  - *Moayyedi et Achkar 2006*

Résultat « net » du dépistage du cancer colorectal avec **RSOS biannuel** chez 1000 individus de 60 ans suivis pendant 12-13 ans

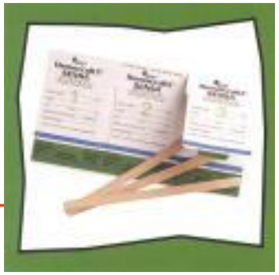

(+)

- 1-2 ont leur vie allongée (?)
- Être rassuré (?)
  - 950 dépistage -

(-)

- Être rassuré à tort
  - 3 cancers manqués
- Être inquiété à tort
  - 50 coloscopies de plus
    - 44 sans cancer
    - <1 avec complications
- Être traité à tort (?)

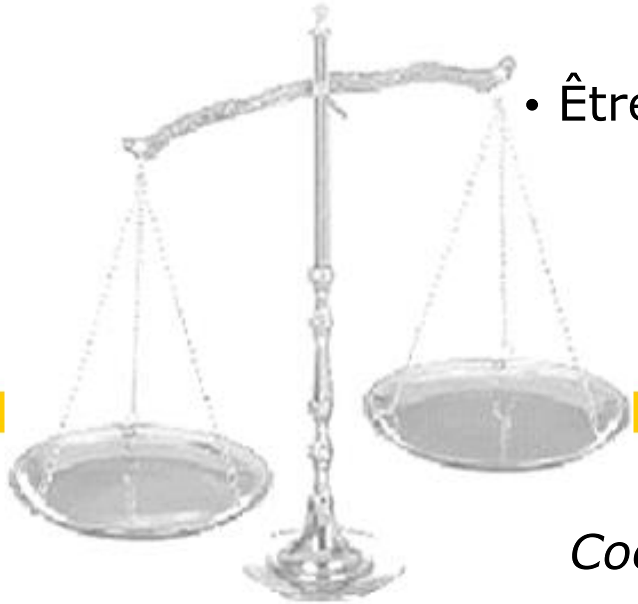

UNIVERSITÉ LAVAL

Cochrane 2006; INSPQ 2008

Résultat « net » du dépistage du cancer colorectal avec **RSOS biannuel** chez 1000 individus de 60 ans suivis pendant 12-13 ans

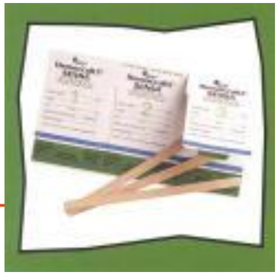

(+)

- 1-2 ont leur vie allongée (?)
- Être rassuré (?)
  - 950 dépistage -

(-)

- Être rassuré à tort
  - 3 cancers manqués
- Être inquiété à tort
  - 50 coloscopies de plus
    - 44 sans cancer
    - <1 avec complications
- Être traité à tort (?)

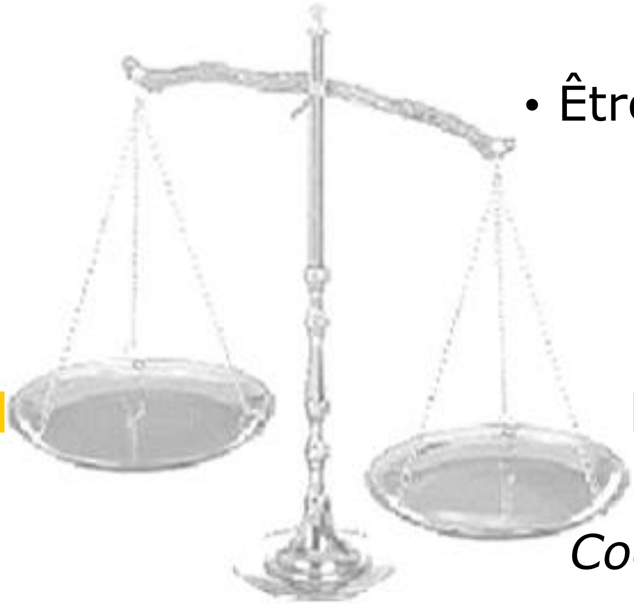

UNIVERSITÉ LAVAL

Cochrane 2006; INSPQ 2008

Résultat « net » du dépistage du cancer colorectal avec une seule sigmoïdoscopie chez 1000 individus de 60 ans suivi pendant 11 ans

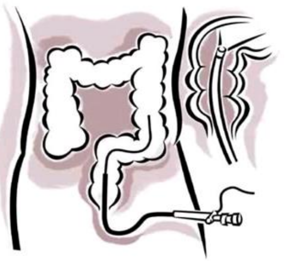

(+)

- 2 ont leur vie allongée (?)
- Être rassuré (?)
  - 950 dépistage –
  - 5 cancers de moins

(-)

- Être rassuré à tort
  - 2 cancers manqués initialement
  - 8 cancers trouvés plus tard
- Être inquiété à tort
  - 50 coloscopies de plus
    - 48 sans cancer
    - <1 avec complications (?)
- Être traité à tort (?)
  - 42 de plus sous surveillance

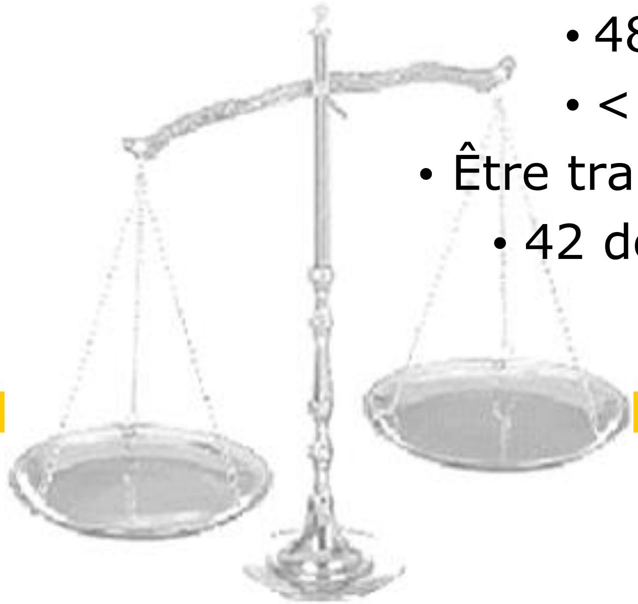

UNIVERSITÉ LAVAL

Atkin et al, Lancet 2010

Résultat « net » du dépistage du cancer colorectal avec **une seule sigmoïdoscopie** chez 1000 individus de 60 ans suivi pendant 11 ans

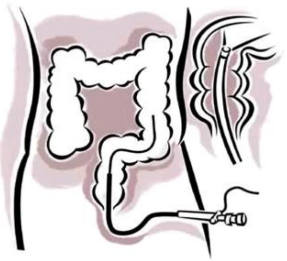

(+)

- 2 ont leur vie allongée (?)
- Être rassuré (?)
    - 950 dépistage –
    - 5 cancers de moins

(-)

- Être rassuré à tort
    - 2 cancers manqués initialement
    - 8 cancers trouvés plus tard
- Être inquiété à tort
    - 50 coloscopies de plus
        - 48 sans cancer
        - <1 avec complications (?)
- Être traité à tort (?)
    - 42 de plus sous surveillance

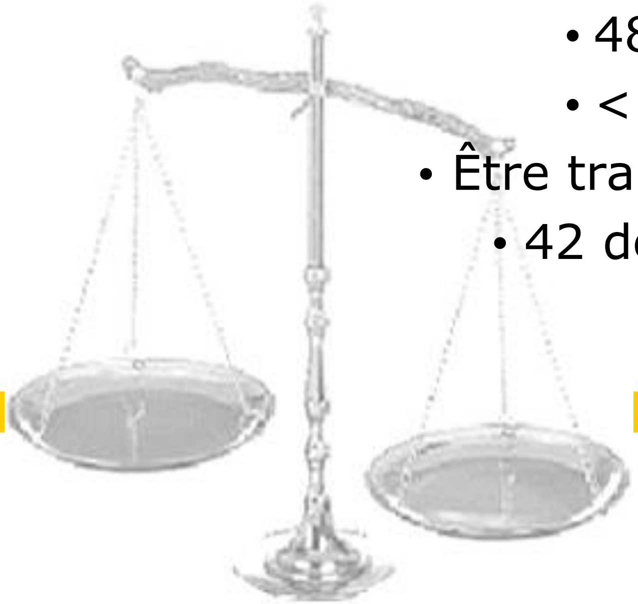

UNIVERSITÉ LAVAL

Atkin et al, Lancet 2010

PERTINENCE ET FAISABILITÉ D'UN PROGRAMME DE DÉPISTAGE DU CANCER COLORECTAL AU QUÉBEC

Rapport du comité scientifique constitué par l'Institut national de santé publique du Québec

OCTOBRE 2008

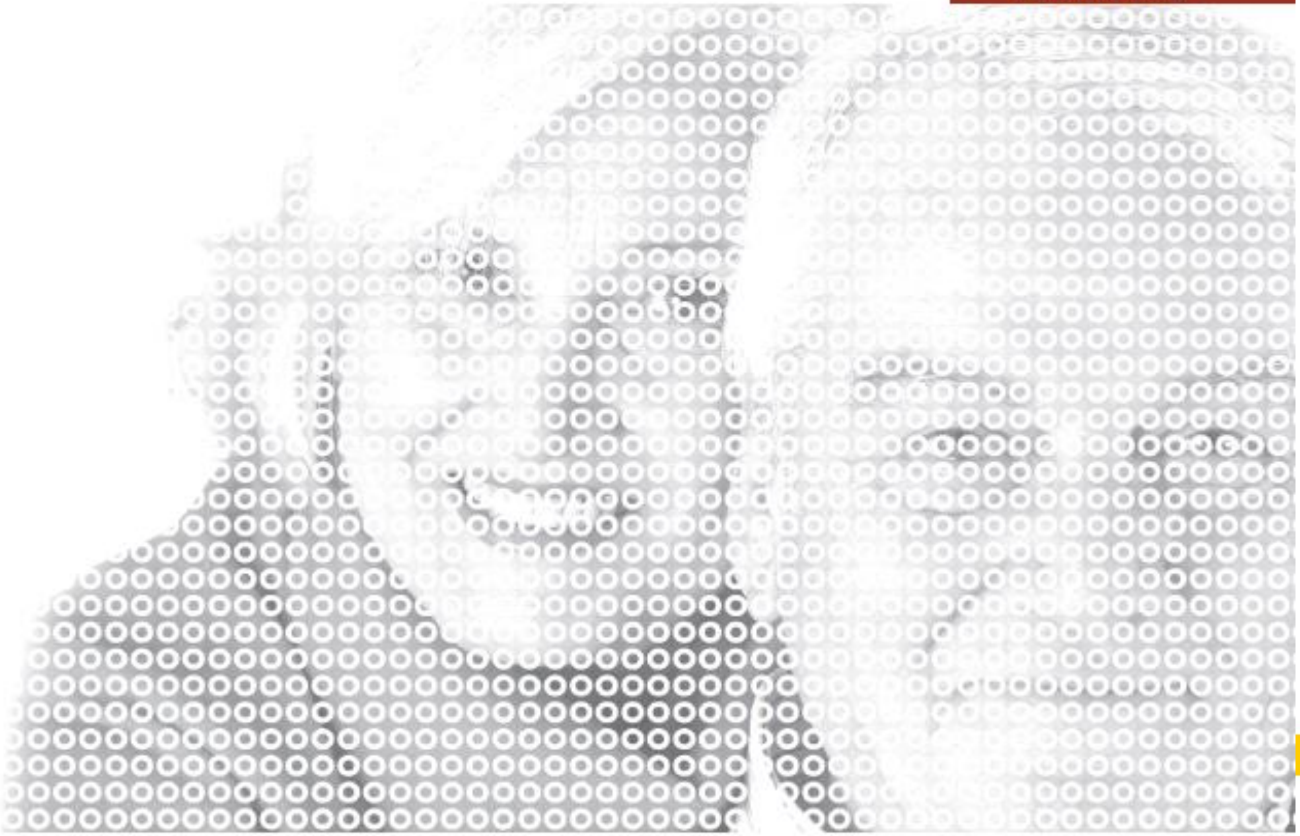

Institut national de santé publique du Québec

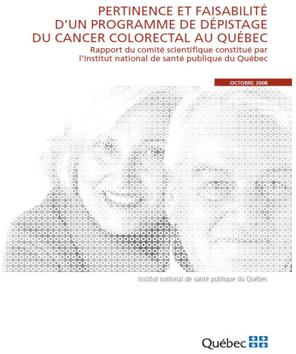

Les recommandations

- Assurer l'accessibilité à la coloscopie

50 ans: 100 000 à 135 000/an
50 à 74 ans: 2 351 000

Efficacité similaire à la sigmoïdoscopie flexible
Neugut et Lebwohl Jama 2010

Projets pilotes anglais et australien
- pas de respect des délais cibles
- délais d'attente hors pilote x 2-3

UNIVERSITÉ LAVAL

INSPQ 2008

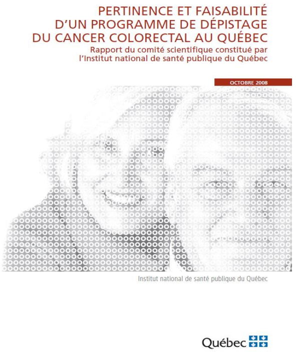

Les recommandations

- Assurer l'accessibilité à la coloscopie
- Priorité aux clientèles à haut risque
  - histoire personnelle
  - histoire familiale
  - maladies inflammatoires de l'intestin
  - cancer colorectal héréditaire sans polypose (Syndrome de Lynch)
  - Polypose adénomateuse familiale

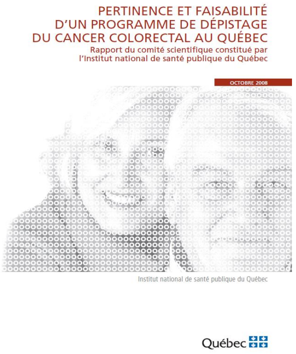

Les recommandations

- Assurer l'accessibilité à la coloscopie
- Priorité aux clientèles à haut risque
- Projet pilote pour les personnes à risque moyen
  - 50 à 74 ans
  - RSOS tous les 2 ans
    - gaïac *vs* immunochimique
  - Informations, outils et éventuellement soutien pour prendre une décision éclairée
  - sans passer par un médecin de famille

Cancer du sein

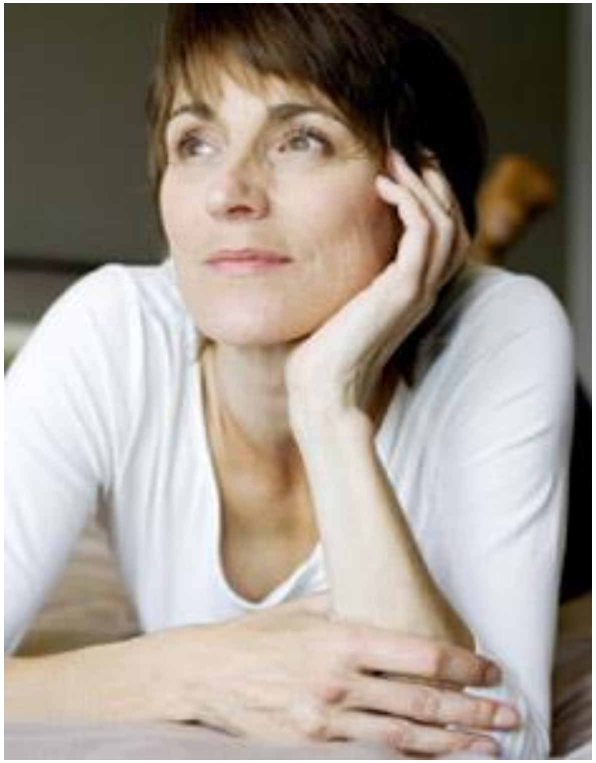

Vivre plus longtemps grâce au dépistage ?

1000 femmes de **50 ans** de votre clientèle ont participé pendant **10 ans** au **programme national** de dépistage du **cancer du sein**. Combien ont eu leur vie allongée au-delà de ces 10 ans grâce à ce programme?

- ❑ Aucune
- ☑ 2
- ❑ 15
- ❑ 34
- ❑ 112

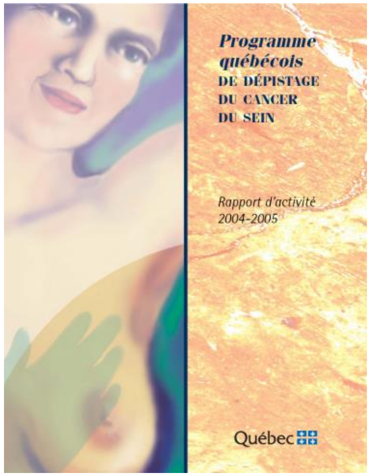

Résultat « net » du dépistage du cancer du sein chez 1000 femmes de 50 ans pendant 10 ans

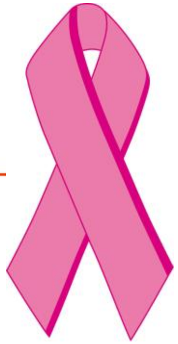

(+)

- 2 ont « la vie sauve » (?)
  - 7 à vie (?)
- Être rassurée (?)
  - 748 mammographie -
  - Mais 10 ont un cancer d'intervalle

(-)

- Être inquiétée à tort (?)
  - 219 sur 242 mammographie + n'ont pas de cancer
  - 64 ont une biopsie
- Être traitée à tort
  - 11 Dx et Rx de plus (?)
    - 5 à vie (?)

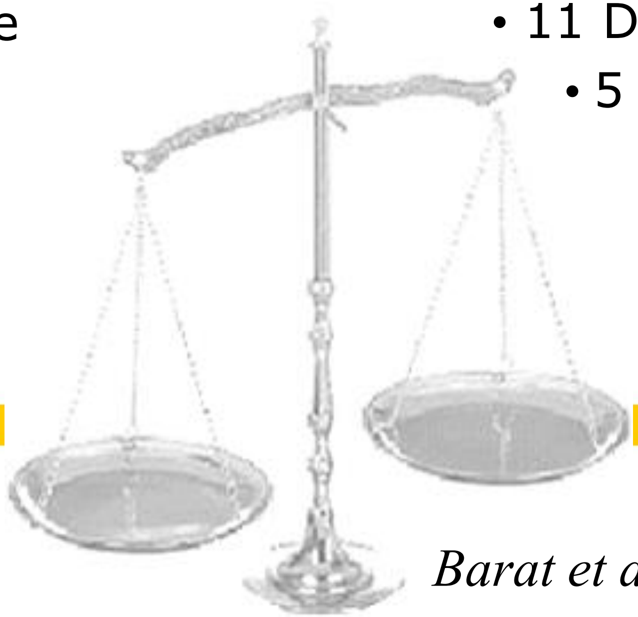

UNIVERSITÉ LAVAL

Barat et al. BMJ 2005, Cochrane 2006

Résultat « net » du dépistage du cancer du sein chez 1000 femmes de 50 ans pendant 10 ans

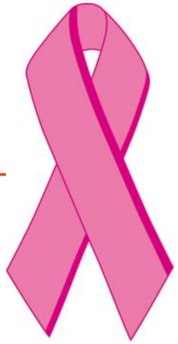

(+)

- 2 ont « la vie sauve » (?)
  - 7 à vie (?)
- Être rassurée (?)
  - 748 mammographie -
  - Mais 10 ont un cancer d'intervalle

(-)

- Être inquiétée à tort (?)
  - 219 sur 242 mammographie + n'ont pas de cancer
  - 64 ont une biopsie
- Être traitée à tort
  - 11 Dx et Rx de plus (?)
    - 5 à vie (?)

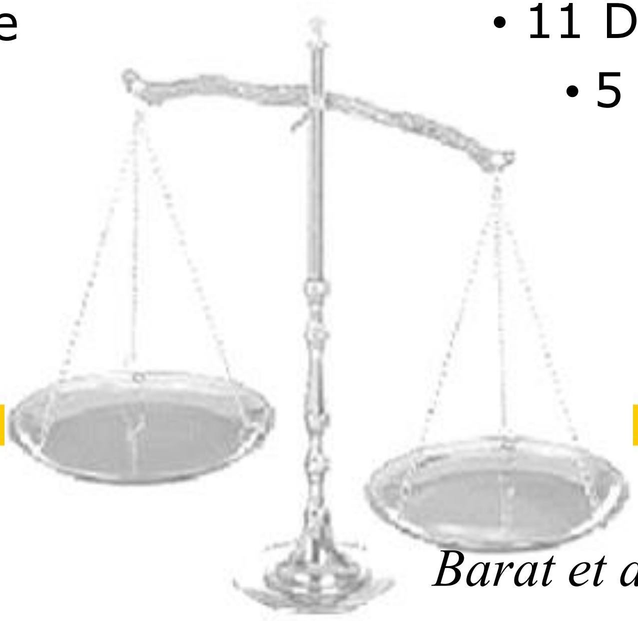

UNIVERSITÉ LAVAL

Barat et al. BMJ 2005, Cochrane 2006

La validité de l'information est incertaine

- « La validité de la plupart des essais… suscite des réserves en raison des nombreuses faiblesses méthodologiques… Des programmes de dépistage modernes et bien menés **pourraient** permettre de détecter et de diagnostiquer le cancer du sein à un stade plus précoce et **peut-être** obtenir des réductions de la mortalité… »

AETMIS 2005

- « Il n'est **pas clair** que le dépistage fasse plus de bien que de mal.

Cochrane 2009

- «Pour le dépistage avec la mammographie aux deux ans chez les femmes de 50 à 74 ans, il y a une **certitude modérée** qu'il existe un **bénéfice net modéré**. »

USPSTF 2009

Participer au PQDCS, un choix qui vous appartient

Le dépista[illegible] : des chiffres pour appuyer votre décision

Pour vous a[illegible]er, voici une comparaison entre un groupe de 1 000 femmes âgées de 50 ans qui participeraient [illegible] dépistag[illegible] pendant 20 ans, soit jusqu'à l'âge de 69 ans, et un groupe de 1000 femmes qui ne participeraient pas a[illegible] dépist[illegible]e. Participer au Programme signifie passer une mammographie de dépistage tous les deux ans.

Vo[illegible] ce qui arrive	1000 femmes participent au Programme de dépistage	1000 femmes n'ayant jamais eu de mammographies dans le cadre du Programme de dépistage
1-Moins de décès par cancer du sein chez les participantes	13	20
2-Plus de cancers du sein découverts chez les participantes	66	50
3-Plus de mammographies et d'échographies pour diagnostiquer une anomalie chez les participantes	925	250
4-Plus de biopsies pour diagnostiquer une anomalie chez les participantes	185	50
5-Des cas de cancer du sein traités, mais qui n'auraient pas eu d'impact sur la vie de la personne (surdiagnostic)	12	0
6-Un risque de décès dû aux radiations associées à la mammographie	0,3	0

L'information présentée dans le tableau provient de: Agence d'évaluation des technologies et des modes d'intervention en santé (AETMIS). *Mammographie de dépistage chez les femmes de 40 à 49 ans : mise à jour.* Rapport préparé par Wilber Deck. ETMIS 2009; 5 (8) : 1-50

En résumé, en participant au dépistage :

- Plus de chance d'éviter une chirurgie majeure, comme l'ablation complète d'un sein à cause d'un cancer; Certains décès par cancers du sein vont être évités;
- Certains cancers du sein seront diagnostiqués et traités pour rien (surdiagnostic);
- [illegible]us d'examens (mammographies, échographies, biopsies) seront nécessaires à cause des anomalies trouvées a[illegible] dep[illegible]stage.

On ne pe[illegible] pas dire si vous serez parmi celles qui allongeront leur vie ou parmi celles qui auront un traitement [illegible]utile. La décision d[illegible] participer ou non au dépistage dépend de la valeur personnelle que vous accordez à ces av[illegible]tages et à ces inconvénie[illegible].

AVERTISSEMENT : L'info[illegible]tion contenue dans ce dépliant ne remplace pas l'a[illegible] de votre médecin. Elle ne devrait pas contribuer à retarder des [illegible]mens ou des traitements que vot[illegible]decin pourrait recommander.

Si vous avez un des symptômes suivants, consultez votre médecin sans tarder, surtout si le symptôme est apparu ou s'est aggravé récemment :
- une bosse à l'intérieur d'un sein;
- un écoulement du mamelon (un seul côté, de façon spontanée);
- l'inversion récente d'un mamelon;
- tout changement (rougeur, pli, rétraction, rides, eczéma, etc.) de la peau du sein ou du mamelon;
- tout changement dans l'apparence d'un sein.

Dans ces cas, la mammographie de dépistage n'est peut-être pas un examen suffisant pour vous.

Version de travail

Vous êtes invitée à participer au Programme québécois de dépistage du cancer du sein (PQDCS).

Si vous décidez d'y participer, vous bénéficiez des avantages suivants :
- Vous pourrez utiliser la lettre d'invitation ou l'ordonnance de votre médecin pour prendre votre rendez-vous pour la mammographie de dépistage.
- Vous et votre médecin recevrez chacun le résultat de votre mammographie de dépistage par envoi postal.
- Si vous devez subir d'autres examens, le responsable du Programme de votre région s'assurera qu'un médecin prend votre suivi médical en charge.
- Vous passerez votre mammographie dans un centre [illegible]e dépistage qui satisfait aux normes de qualité du P[illegible]ogramme.
- V[illegible]us participerez à un programme dont les résultats so[illegible]t évalués régulièrement et publiés sur le site ww[illegible].inspq.qc.ca/groupes/pqdcs.
- Vou[illegible] recevrez une invitation à passer une mammographie de d[illegible]pistage une fois tous les deux ans.

Si vou[illegible] souhaitez passer une mammographie, mais n[illegible]e voulez pas participer au PQDCS, que se passe[illegible]-t-il ?

La ma[illegible]mographie de dépistage vous est quand même offerte[illegible] sans frais dans tous les centres de dépistage recon[illegible]us du PQDCS. Toutefois, si vous décidez de ne pas p[illegible]rticiper au Programme, vous devrez signer votre refu[illegible] sur le formulaire de consentement au Programme. Cel[illegible] signifie aussi que :
- [illegible]ous aurez besoin d'une ordonnance de votre médecin chaque fois que vous voudrez passer une mammographie de dépistage.
- Votre médecin recevra le résultat de votre mammographie et sera responsable de faire le suivi médical requis.
- Les renseignements à votre sujet ne serviront pas à évaluer et à améliorer la performance du PQDCS.
- Vous ne recevrez plus de lettre d'invitation tous les deux ans vous rappelant qu'il est temps de passer votre mammographie.

Vous avez décidé de ne pas participer, mais aimeriez réintégrer le Programme quelques années plus tard?

Vous pouvez toujours revenir au PQDCS. Pour réintégrer le Programme vous devrez :
- Obtenir une ordonnance de mammographie de dépistage de votre médecin.
- Signer le formulaire de consentement à participer au Programme lors de votre mammographie dans un centre de dépistage désigné.

Santé et Services sociaux Québec

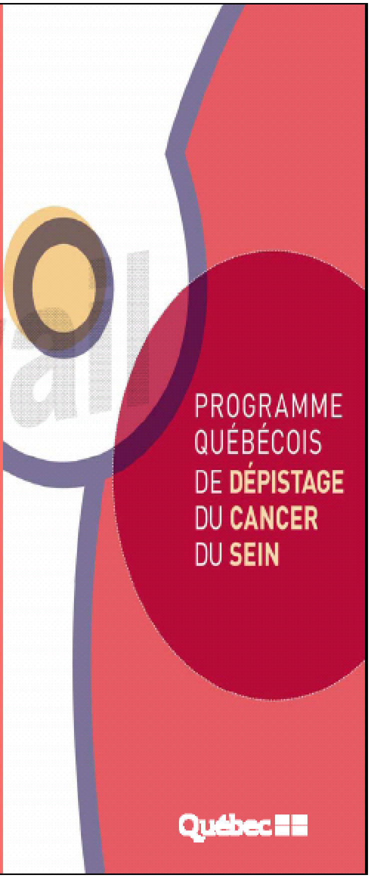

UNIVERSITÉ LAVAL

Favoriser la participation active du patient à la prise de décision

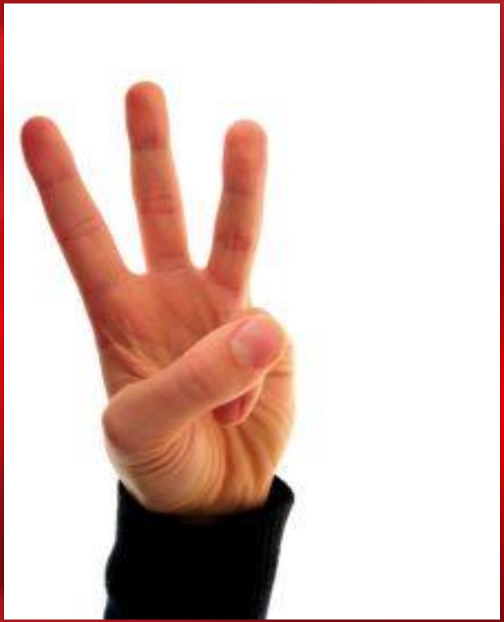

UNIVERSITÉ LAVAL

Qu’est-ce que vous en avez retenu?

Jean-Claude, avez-vous l’impression d’avoir bien compris ce dont je viens de vous parler?

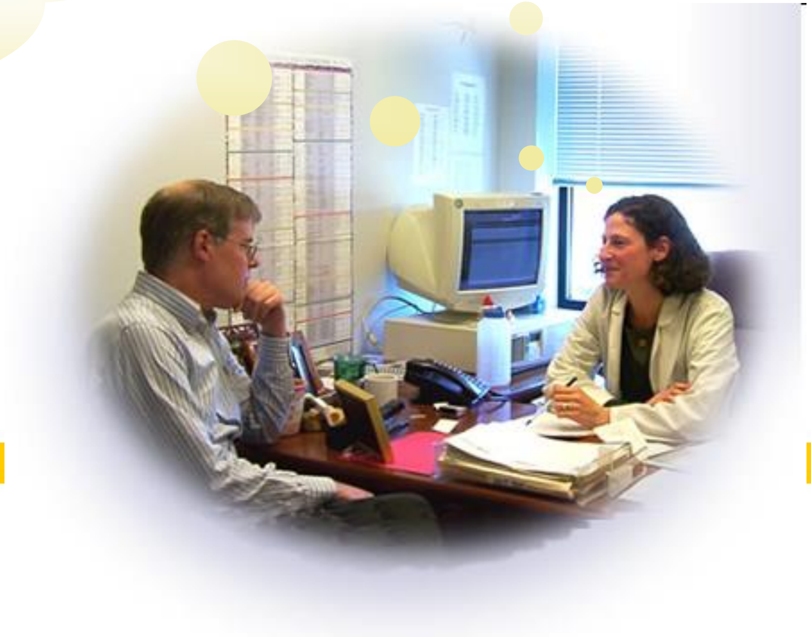

Rechercher les valeurs et préférences du patient

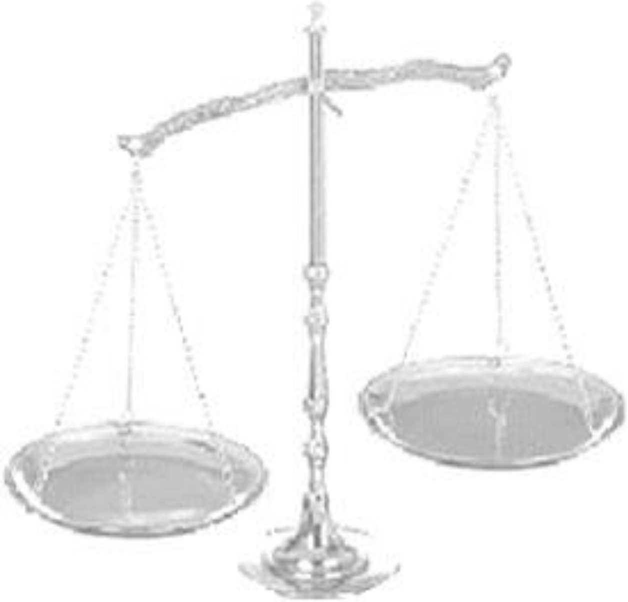

UNIVERSITÉ LAVAL

Marie, avez-vous le sentiment d'avoir toute l'information sur les bons et les moins bons côtés du dépistage?

Qu'est-ce qui est le plus important pour vous ? Augmenter vos chances d'allonger votre vie grâce au dépistage ou ne pas courir le risque des limites et des inconvénients du dépistage ?

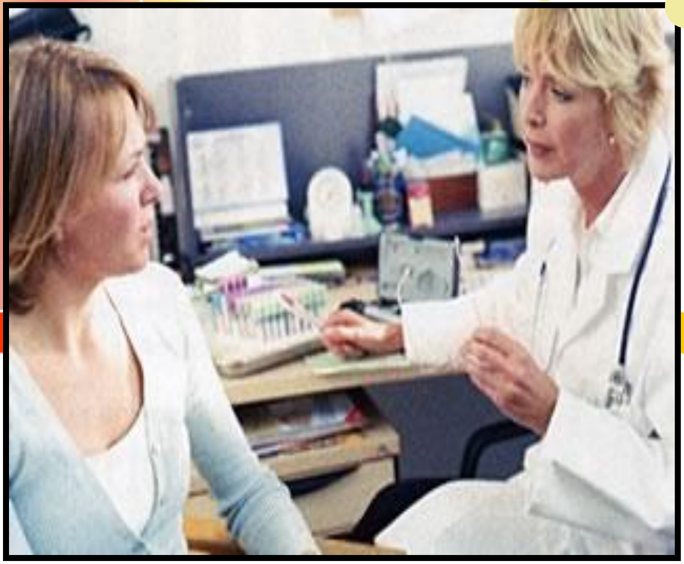

Est-ce qu'il y a d'autres choses importantes pour vous qui vous inciteraient à faire ou à ne pas faire le dépistage?

Êtes-vous certain Jean-Claude que c'est ce qui est le plus important pour vous?

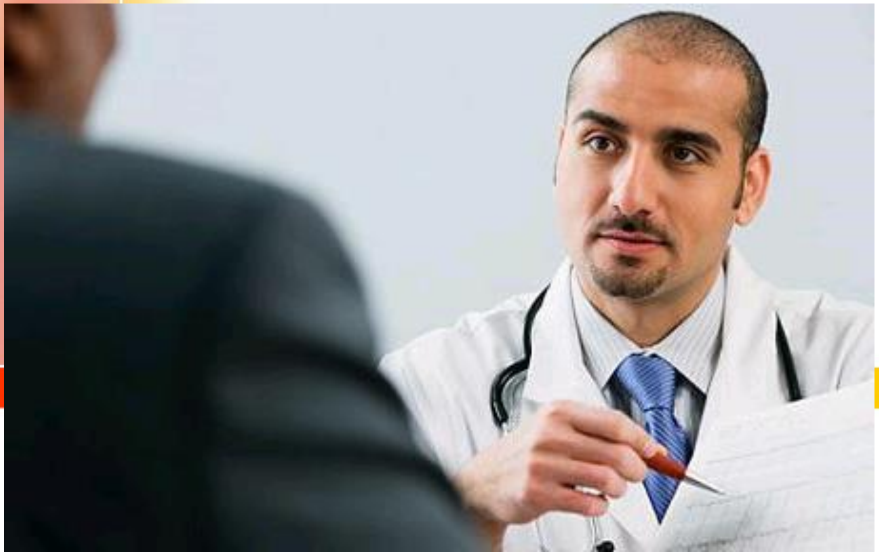

S’assurer que le patient est confortable avec sa décision

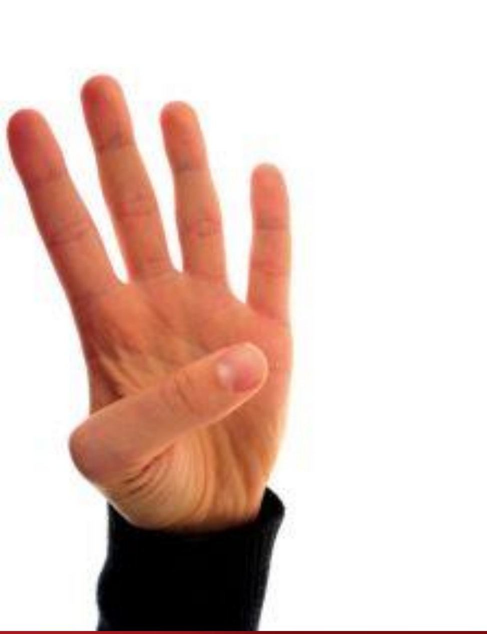

Évaluer le confort décisionnel du patient (S.U.R.E.)

		OUI	NON	Ne sais pas
**Sûr(e) de moi…**	1) Je suis certain/e du meilleur choix pour moi.	☑	☐	☐
**Utilité de l'information…**	2) J'ai le sentiment d'avoir toute l'information nécessaire sur les bons et moins bons côtés de toutes les options disponibles.	☑	☐	☐
**Risques et bénéfices à balancer…**	3) J'ai le sentiment de savoir ce qui est le plus important pour moi à l'égard des risques/bénéfices qui sont associés à chacune des options disponibles.	☑	☐	☐
**Encouragement et soutien des autres…**	4) J'ai tout le soutien dont j'ai besoin pour faire mon choix.	☑	☐	☐

UNIVERSITÉ LAVAL

O'Connor et Légaré 2006

Avez-vous le sentiment de pouvoir prendre une décision maintenant?

Désirez-vous y réfléchir un peu et qu’on en reparle?

Êtes-vous certaine de votre décision?

Avez-vous le sentiment que vos proches seront d’accord avec votre décision?

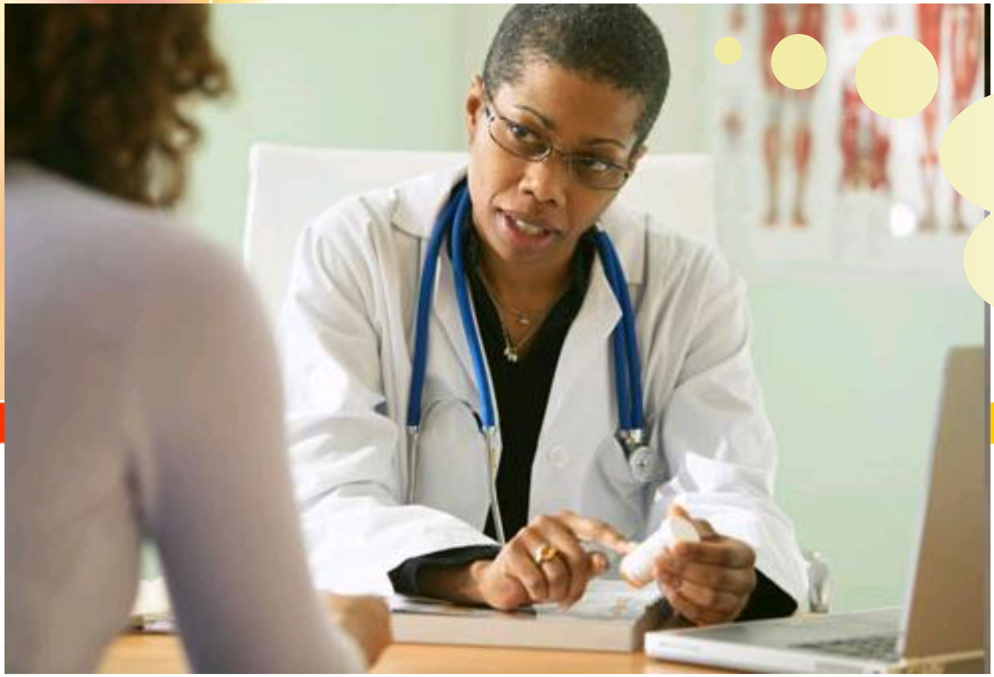

Les 4 étapes pour intégrer la prise de décision partagée en pratique

1. Initier un processus de prise de décision partagée

2. Partager de façon claire et balancée les informations pertinentes

   L'information sur les ***bénéfices*** *potentiels doit être* ***mise en balance*** *avec celle sur les* ***risques*** *pour décider de faire ou non le dépistage.*

UNIVERSITÉ LAVAL

Les 4 étapes pour intégrer la prise de décision partagée en pratique (suite)

3. Favoriser la participation active du patient à la prise de décision

   *L'information seule est **insuffisante** pour soutenir la prise de décision d'avoir recours ou non au dépistage.*

4. S'assurer que le patient est confortable avec sa décision

   *Des **outils d'aide à la décision** sont utiles (voire essentiels!) pour aider à prendre une décision éclairée.*

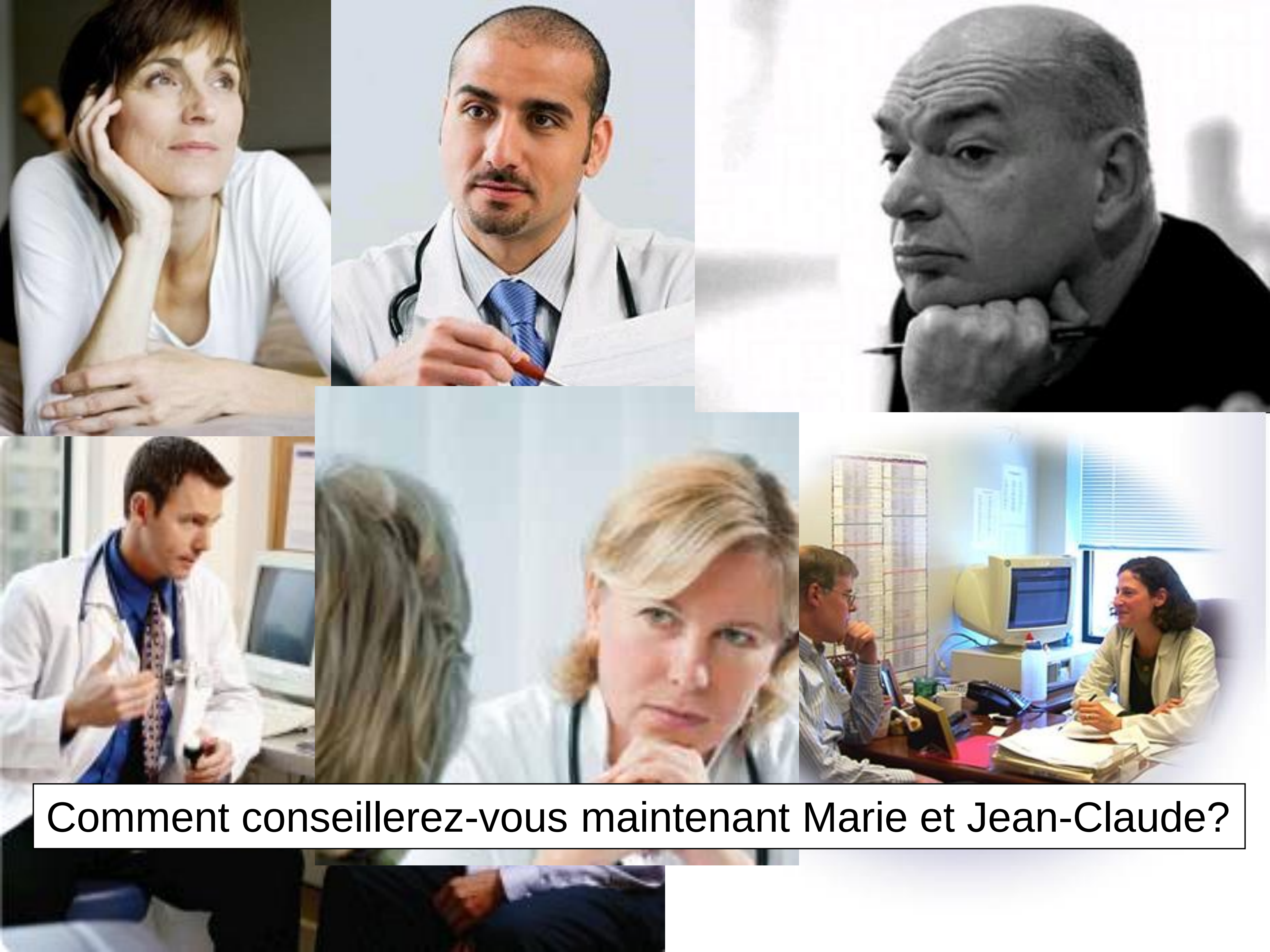

Comment conseillerez-vous maintenant Marie et Jean-Claude?

Le programme Québécois de dépistage du cancer du sein a besoin de votre opinion !

- Le MSSS désire revoir ses outils de communication afin que les femmes ciblées par le PQDCS puissent prendre une décision éclairée sur leur participation au programme de dépistage du cancer du sein.

- Imaginez que cette approche soit en place:

  Quelle est votre intention d'aider les femmes ciblées par le PQDCS à prendre une décision éclairée sur leur participation au dépistage du cancer du sein?
