# Supplementary material for: Family physicians’ intention to support women in making informed decisions about breast cancer screening with mammography: a cross-sectional survey
Source: BMC Res Notes. 2015 Nov 10;8:663. doi: 10.1186/s13104-015-1608-8 (PMC4640384; doi:10.1186/s13104-015-1608-8)
Supplement: Supplementary file 2 — 10.1186/s13104-015-1608-8 Questionnaire (translated from the original in French). [file 13104_2015_1608_MOESM2_ESM.docx]

**Additional File 2 – Questionnaire (translated from the original in French)**

For the past 10 years, the Quebec Ministry of Health and Social Services (MHSS) has invited all women aged 50 to 69 by letter to participate in the Quebec Breast Cancer Screening Program (QBCSP). Until recently the QBCSP encouraged all women in this age group to participate in the program. The MHSS is considering changing this approach.

In line with its current slogan "Screening for breast cancer: a decision that belongs to you," the QBCSP intends to put in place mechanisms aimed at informing women about the potential benefits as well as inconveniences of breast cancer screening, based on the most valid scientific evidence. More specifically, this will consist of awareness campaigns targeting the general public and health professionals, information leaflets, a website for women invited to participate in the program, and training programs for health professionals on informed decision making about breast cancer screening.

The goal is to ensure that every woman invited by the QBCSP is able to make informed decisions about whether to undergo breast cancer screening or not. According to this approach, there is no best decision (to screen) or worst decision (not to screen). Both options are acceptable. Each woman has to choose the option with which she feels most comfortable after having reviewed the information provided. Now, imagine that this approach has already been adopted, and then please respond to the following questions.

1. I intend to help women targeted by the QBCSP in making informed decisions about screening for breast cancer: (INTENTION)*

| -3 | -2 | -1 | 1 | 2 | 3 |
| --- | --- | --- | --- | --- | --- |
| strongly disagree | | | strongly agree | | |

2. The likelihood that I will help women targeted by the QBCSP in making informed decisions about screening for breast cancer is: (INTENTION)*

| -3 | -2 | -1 | 1 | 2 | 3 |
| --- | --- | --- | --- | --- | --- |
| very weak | | | very strong | | |

3. I feel that helping women targeted by the QBCSP in making informed decisions about screening for breast is:

(ATTITUDE)*

| -3 | -2 | -1 | 1 | 2 | 3 |
| --- | --- | --- | --- | --- | --- |
| useless | | | very useful | | |

(CONTROL)*

| -3 | -2 | -1 | 1 | 2 | 3 |
| --- | --- | --- | --- | --- | --- |
| very difficult | | | very easy | | |

(INTENTION)*

| -3 | -2 | -1 | 1 | 2 | 3 |
| --- | --- | --- | --- | --- | --- |
| very unlikely | | | very likely | | |

(ATTITUDE)*

| -3 | -2 | -1 | 1 | 2 | 3 |
| --- | --- | --- | --- | --- | --- |
| very irresponsible | | | very responsible | | |

4. Most people in my work environment would approve / disapprove of me helping women targeted by the QBCSP in making informed decisions about screening for breast cancer: (SOCIO-PROFESSIONAL NORM)*

| -3 | -2 | -1 | 1 | 2 | 3 |
| --- | --- | --- | --- | --- | --- |
| strongly disapprove | | | strongly approve | | |

5. I don’t see any obstacles in helping women targeted by the QBCSP to make informed decisions about screening for breast cancer: (CONTROL)*

| -3 | -2 | -1 | 1 | 2 | 3 |
| --- | --- | --- | --- | --- | --- |
| strongly disagree | | | strongly agree | | |

6. I consider that there are more advantages than disadvantages for women targeted by the QBCSP if I help them in making informed decisions about screening for breast cancer: (ATTITUDE)*

| -3 | -2 | -1 | 1 | 2 | 3 |
| --- | --- | --- | --- | --- | --- |
| strongly disagree | | | strongly agree | | |

7. Most people who are important to me would approve / disapprove of me helping women targeted by the QBCSP in making informed decisions about screening for breast cancer. (SOCIO-PROFESSIONAL NORM)*

| -3 | -2 | -1 | 1 | 2 | 3 |
| --- | --- | --- | --- | --- | --- |
| would strongly disapprove | | | would strongly approve | | |

8. I am able to help women targeted by the QBCSP in making informed decisions about screening for breast cancer. (CONTROL)*

| -3 | -2 | -1 | 1 | 2 | 3 |
| --- | --- | --- | --- | --- | --- |
| strongly disagree | | | strongly agree | | |

9. Most of my patients targeted by the QBCSP would approve / disapprove of me helping them in making informed decisions about screening for breast cancer: (SOCIO-PROFESSIONAL NORM)*

| -3 | -2 | -1 | 1 | 2 | 3 |
| --- | --- | --- | --- | --- | --- |
| would strongly disapprove | | | would strongly approve | | |

10. Gender: Female: ________ Male: ________

11. Year of issue of license to practice _________________

Your comments and suggestions on the factors that could hinder or facilitate you supporting women in making informed decisions about screening for breast cancer are highly valuable. Thank you for sharing them with us.

12. Factors that could hinder me: ____________________________________________________________________________________________________________________________________________________________________________________________________________________________________________________________________________________________

13. Factors that could help me: ________________________________________________________________________________________________________________________________________________________________________________________________________________________________________________________________________________________________

Comments:______________________________________________________________________________________________________________________________________

Please drop your completed questionnaire in the boxes labelled QBCSP at the door.

THANK YOU FOR YOUR COLLABORATION

* The constructs (intention, attitude, control, and socio-professional norm were not identified in the participants’ questionnaire)
